# Temporal trends of sepsis-related mortality in China, 2006–2020: a population-based study

**DOI:** 10.1186/s13613-023-01166-1

**Published:** 2023-08-14

**Authors:** Run Dong, Wei Liu, Li Weng, Peng Yin, Jinmin Peng, Yan Chen, Shan Li, Chunyao Wang, Wei Jiang, Xiaoyun Hu, Bin Du, Maigeng Zhou, for the China Critical Care Clinical Trials Group (CCCCTG)

**Affiliations:** 1 Medical Intensive Care Unit, State Key Laboratory of Complex Severe and Rare Diseases, Peking Union Medical College Hospital, Peking Union Medical College, Chinese Academy of Medical Sciences, Beijing, China; 2 National Center for Chronic and Noncommunicable Disease Control and Prevention, Chinese Center for Disease Control and Prevention, Beijing, China

**Keywords:** Sepsis, Sepsis-related mortality, Years of life lost, Temporal trends, Sociodemographic index

## Abstract

**Background:**

The scarcity of sepsis epidemiologic data from most low- and middle-income countries (LMICs) hampered estimation of regional and global burden of the disease, and provided limited guidance for policy makers. We aimed to characterize and analyze the temporal trends of sepsis-related mortality in China, by population groups, underlying causes of death, geographic regions, and sociodemographic index (SDI) levels.

**Methods:**

Sepsis-related deaths were identified from the National Mortality Surveillance System (NMSS) of China from 2006 to 2020. Trends of sepsis-related mortality and years of life lost (YLLs), stratified by age, sex, underlying diseases, and regions were analyzed using the Jointpoint regression analysis. We investigated the association of SDI with trends of sepsis-related mortality.

**Results:**

In 2020, sepsis was estimated to be responsible for 986,929 deaths and 17.1 million YLLs in China. Age-standardized sepsis-related mortality significantly declined from 130.2 (95%CI, 129.4–131) per 100,000 population in 2006 to 76.6 (76.3–76.9) in 2020. Age-standardized YLLs decreased from 2172.7 (2169.4–2176) per 100,000 population in 2006 to 1271 (1269.8–1272.2) in 2020. Substantial variations of sepsis-related mortality and YLLs were observed between population groups and regions, with higher burden in males, the elderly, and western China. An inverse relation was noted between SDI and sepsis-related mortality or YLLs.

**Conclusions:**

Despite declining trends of age-standardized mortality and YLLs of sepsis in China, significant disparities between population groups and regions highlight a need for targeted policies and measures to close the gaps and improve the outcome of sepsis.

**Supplementary Information:**

The online version contains supplementary material available at 10.1186/s13613-023-01166-1.

## Background

Sepsis is defined as life-threatening organ dysfunction due to a dysregulated host response to infection [1]. It is a global health threat with high incidence and mortality. The Global Burden of Disease (GBD) Study estimated 48.9 million incident cases of sepsis and 11.0 million sepsis-related deaths worldwide in 2017, representing 19.7% of global deaths [2]. As was emphasized by the World Health Organization (WHO) Global Report on the Epidemiology and Burden of Sepsis, epidemiological research of sepsis is critical to inform interventions for its prevention, diagnosis and management considering its serious consequences and preventable nature. [3]

However, the scarcity of sepsis epidemiologic data from most low- and middle-income countries (LMICs) hampered calculations and understanding of global estimates [2, 4, 5]. In China, epidemiological studies of sepsis were limited to hospital-based approach that did not cover the whole population or capture sepsis cases occurring outside the hospital setting [6–8]. To address these limitations, we used vital registration death records from the National Mortality Surveillance System (NMSS) of China. Previously, a population-based analysis of the NMSS reported a standardized sepsis-related mortality of 66.7 per 100,000 population, producing a national estimate of 1,025,997 sepsis-related deaths in 2015 [9]. Based on the NMSS and a public hospital inpatient discharge abstract database, a recent study estimated an annual standardized incidence of hospitalized sepsis of 328.25–421.85 cases per 100,000 population in China from 2017 to 2019 [10].

Nevertheless, no study has characterized temporal trends of sepsis-related mortality in China. The nation has been undergoing rapid transition of cause-of-death patterns from infectious diseases to noncommunicable causes, with large gaps between population groups and provinces [11]. Investigations into trends of sepsis-related mortality and their demographic variations are implicative for the response of the healthcare system to the challenges posed by epidemiological shifts and diverse needs across China.

The sociodemographic index (SDI), developed by GBD researchers, is a composite indicator constructed from measures of per capita income, average years of education, and total fertility rates [12]. The SDI identifies, where countries or regions sit on the spectrum of development, and is associated with many population health indicators [13, 14]. Understanding the relationship between sociodemographic level and sepsis-related mortality may provide evidence for interventions of priority to reduce health loss of sepsis.

Based on the NMSS, we conducted a study to characterize temporal trends of sepsis-related mortality at national and provincial levels of China from 2006 to 2020, stratified by sex, age, and underlying diseases. Sepsis burden was also quantified by years of life lost (YLLs). In addition, we analyzed the association between SDI and subnational disparities of sepsis-related death.

## Methods

### Data source

Mortality data were derived from the NMSS, which covers 323.8 million population (24.3% of the total population of China) and comprises 605 disease surveillance points (DSPs) across all 31 provinces, municipalities, and autonomous regions in China [15]. Representativeness of the NMSS data was achieved by the following procedures. First, at least 5 million or 20% of the total population in each province were under surveillance. Second, surveillance points were selected from each of 8 strata of counties and districts divided according to the degree of urbanization, population size and crude mortality rate in each province. Third, representativeness of surveillance points was evaluated using four parameters, i.e., urbanization index, ratio of population 65 years or older, ratio of population 15 years or younger, and crude mortality rate. An iterative process was conducted until there was no significant difference between the combination of selected surveillance points and the whole province for each given parameter.

The completeness and accuracy of data from NMSS was ensured by annual quality control meetings, staff training courses, development of regulations for death registration, and site quality inspection, following the strategies of the World Health Organization [16].

We collected information of all death certificates reported to the NMSS from 2006 to 2020, including immediate or intermediate causes of death, underlying causes of death (UCD), and demographic characteristics (Additional file 1: Table S1). We also recorded the place, where causes of death were determined, because families might transfer hospitalized patients with impending death back home. Population data were collected from the China National Bureau of Statistics [17]. SDI data were obtained from the Global Burden of Disease Study 2019 [18].

### Definitions

We adopted the approach used and validated in previous studies that presumed death attributed to infection as sepsis-related [9, 19–21]. Sepsis-related mortality was identified in NMSS if any International Classification of Diseases 10th (ICD-10) revision code of acute infection was listed among immediate or intermediate causes of death in part I of death certificates (Additional file 1: Table S2). ICD-10 codes of infection were derived from the modified Angus criteria [22] with the addition of codes of chronic bronchitis (J40, J41, J41.0, J41.1, J41.8, J42, J44.0, J44.1), which probably indicated an infectious cause of acute exacerbation of chronic bronchitis. Underlying causes of sepsis-related deaths are reported according to the underlying GBD causes, 2020 Year book of health in the People’s Republic of China, and National Standard Classification and Codes of Diseases (Additional file 1: Table S3) [18, 23, 24].

### Mortality and YLLs

Sepsis-related mortality was estimated at national and provincial levels from 2006 to 2020, stratified by sex, age, underlying diseases, and place of death. Age-standardized mortality was calculated using the direct method of standardizing to the population of 2020 China Census. YLLs at national and provincial levels were estimated by multiplying the counts of death in each age group by the standard remaining life expectancy at the age of death. We used Poisson regression to estimate 95% confidence intervals (CIs) for sepsis-related deaths, mortality, and YLLs at national and provincial levels.

### Analysis of temporal trends

Trends of age-standardized mortality were tracked using the Jointpoint regression analysis and reported as average annual percentage changes (AAPCs) with 95%CIs [25]. A trend was considered decreasing if AAPC was negative and the 95%CI did not include zero. Positive AAPC and 95%CI indicated increasing trend.

### Correlation analysis of sepsis burden and SDI

Ranging from 0 to 100, the SDI is a composite average of lag-distributed income per capta, average years of education for those aged 15 and older, and total fertility rate among women younger than 25 years. Based on SDI from 1990 to 2019, simple linear regression was used to predict the SDI in 2020. We analyzed the correlation between sepsis-related mortality and SDI from 2006 to 2020 using Spearman’s rho coefficients. Trends of age-standardized sepsis-related mortality and YLLs at provincial level were compared with expected levels based on SDI.

### Statistical analysis and data visualization

Statistical analyses were conducted using the R program (version 4.0.2; R Foundation for Statistical Computing) and Joinpoint software (version 4.9.0.0. March 2021; Statistical Research and Applications Branch, National Cancer Institute). Data visualization was performed using GraphPad Prism (version 9.0; GraphPad Software, Inc., USA), QGIS (version 3.22; QGIS Geographic Information System), and R program (version 4.0.2; R Foundation for Statistical Computing).

## Results

From 2006 to 2020, there were 2,371,274 sepsis-related deaths reported to the NMSS, representing 12.4% of total deaths. Sepsis was estimated to be responsible for 986,929 deaths and 17.1 million YLLs in 2020 nationally. Age-standardized sepsis-related mortality decreased from 130.2 (95%CI 129.4–131) per 100,000 population in 2006 to 76.6 (76.3–76.9) in 2020 (AAPC, − 4.0%; 95%CI − 5.5% to − 2.5%). Age-standardized YLLs of sepsis declined from 2172.6 (2169.4–2176) per 100,000 population in 2006 to 1271 (95%CI, 1269.8–1272.2) in 2020 (AAPC, − 3.7%; 95%CI − 5% to − 2.5%) (Table 1 and Additional file 1: Table S4).

**Table 1:** Number of deaths, age-standardized mortality and YLLs related to sepsis in China, for all ages, both sexes, 2006–2020

	2006	2020	Percentage change (%) or AAPC (%, 95%CI), 2006–2020
Age-standardized mortality per 100,000 population (95%CI)			
Both sexes	130.2 (129.4–131)	76.6 (76.3–76.9)	− 4 (− 5.5 to − 2.5)
Male	161.3 (160.1–162.6)	101.6 (101.1–102)	− 2.3 (− 6.3 to 1.8)
Female	106.6 (105.5–107.6)	56 (55.6–56.3)	− 4.6 (− 6.1 to − 3.1)
Number of deaths (95%CI), national estimate			
Both sexes	834487 (827141–841833)	986929 (982960–990898)	18.3
Male	466370 (460866–471874)	588653 (585582–591723)	26.2
Female	368514 (363643–373385)	398830 (396310–401349)	8.2
Age-standardized YLLs per 100,000 population (95%CI)			
Both sexes	2172.7 (2169.4–2176)	1271 (1269.8–1272.2)	− 3.7 (− 5 to − 2.5)
Male	2707.9 (2702.8–2713)	1716.6 (1714.7–1718.6)	− 3.3 (− 4.6 to − 2)
Female	1715.3 (1711.2–1719.5)	868.8 (867.4–870.2)	− 4.6 (− 5.9 to − 3.4)
YLLs (95%CI), national estimate			
Both sexes	18430833 (18299943–18561723)	17106784 (17046559–17167009)	− 7.2
Male	10879159 (10778968–10979350)	10948986 (10900837–10997135)	0.6
Female	7565565 (7482067–7649063)	6172321 (6136662–6207979)	− 18.4

*95%CI* 95% confidence interval, *AAPC* average annual percentage change, *YLLs* years of life lost

**Fig. 1:**
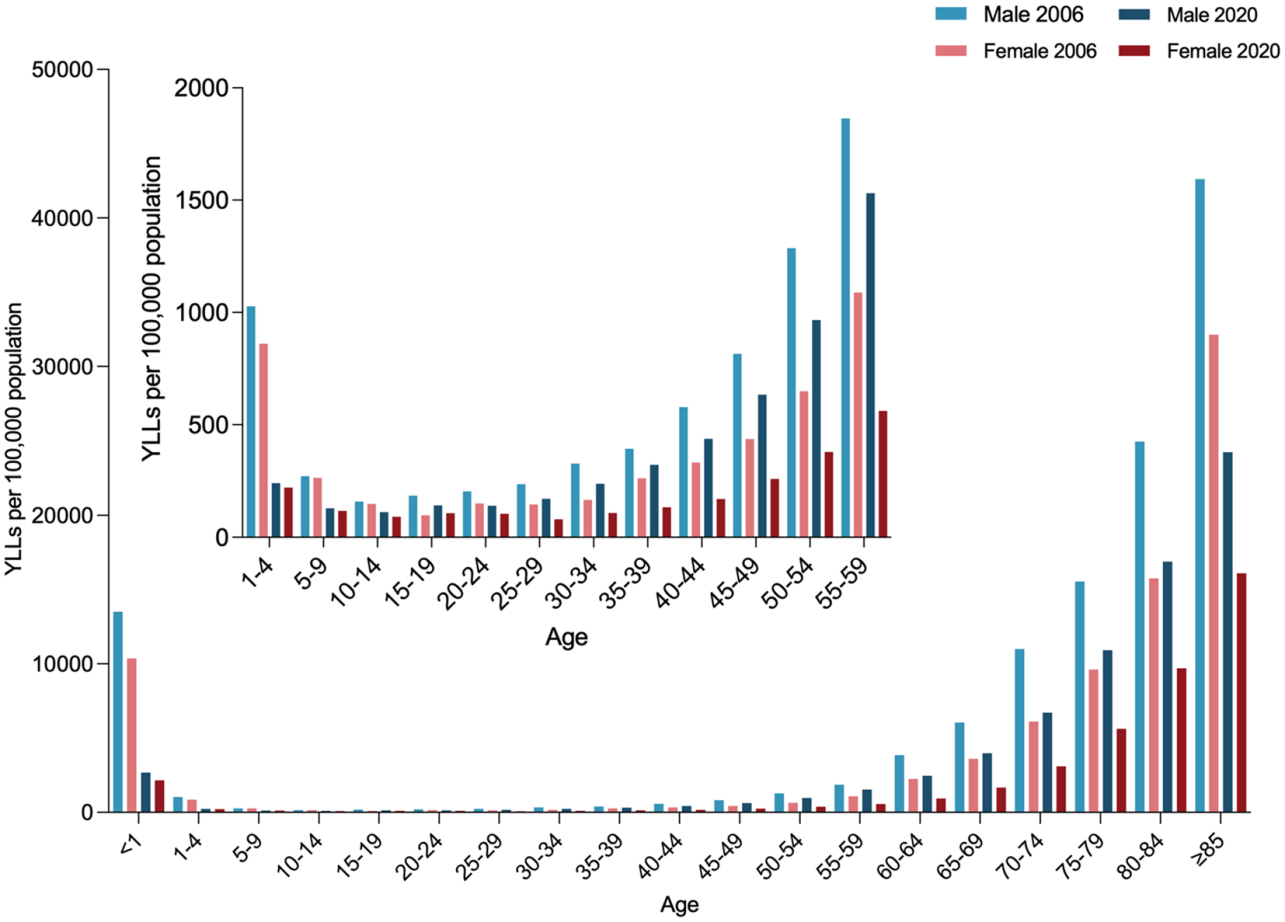
Sepsis-related YLLs per 100,000 population in China, 2006–2020, by sex and age. *YLLs* years of life lost

Age-standardized mortality and YLLs of sepsis was higher among male than female throughout the study period (Table 1 and Additional file 1: Table S4). Trends of sepsis-related mortality remained stable for male (AAPC, − 2.3%; 95%CI − 6.3% to 1.8%), and decreased among female (AAPC, − 4.6%; 95%CI − 6.1% to − 3.1%). Decreasing trends of sepsis-related YLLs were noted among both sexes (AAPC, − 3.3% [95%CI − 4.6% to − 2%] for male and − 4.6% [− 5.9% to − 3.4%] for female). The gender difference was observed in most age groups (Fig. 1 and Additional file 1: Table S5), provinces, and underlying causes of death (data not shown).

**Fig. 2:**
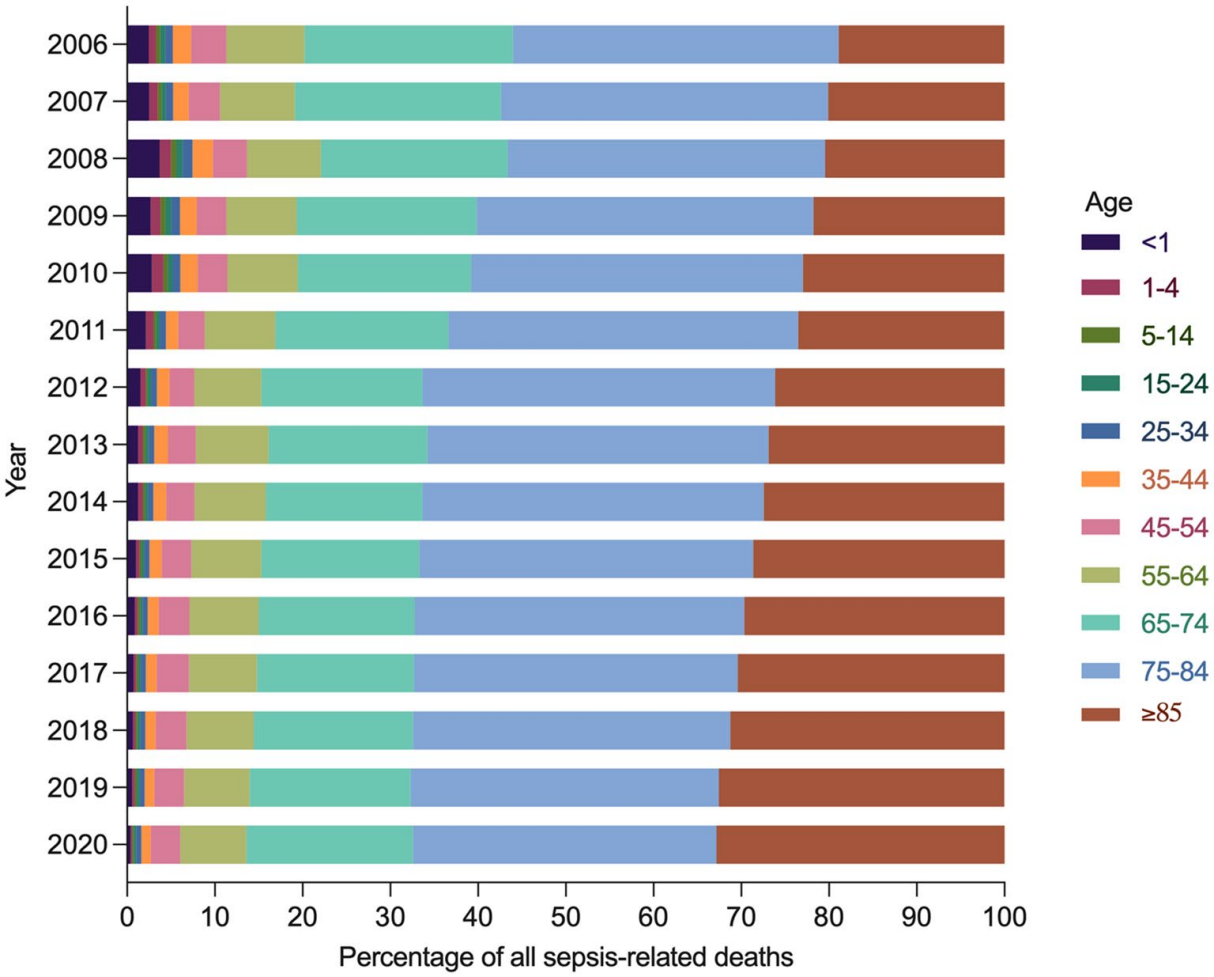
Percentage of all sepsis-related deaths for both sexes in China, 2006–2020, by age

Sepsis caused more deaths in the elderly, especially those over 75 years, compared with other age groups. The proportion of decedents aged 85 years and over increased to 32.8% of all sepsis-related deaths during the study period (Fig. 2 and Additional file 1: Fig. S1). In most age groups, sepsis-related mortality and YLLs declined, with the largest decreases noted among those under 1 year (Fig. 1 and Additional file 1: Table S5). In males, sepsis-related mortality decreased significantly in age groups under 1 year and 55–84 years, and remained stable in other age groups. In females, sepsis-related mortality was unchanged in age groups 1–4 years and greater than 85 years, and decreased significantly in all the other age groups. For both sexes, sepsis-related YLLs declined through childhood, remained low in early adulthood, increased in older adults, and reached the peak among the elderly.

**Fig. 3:**
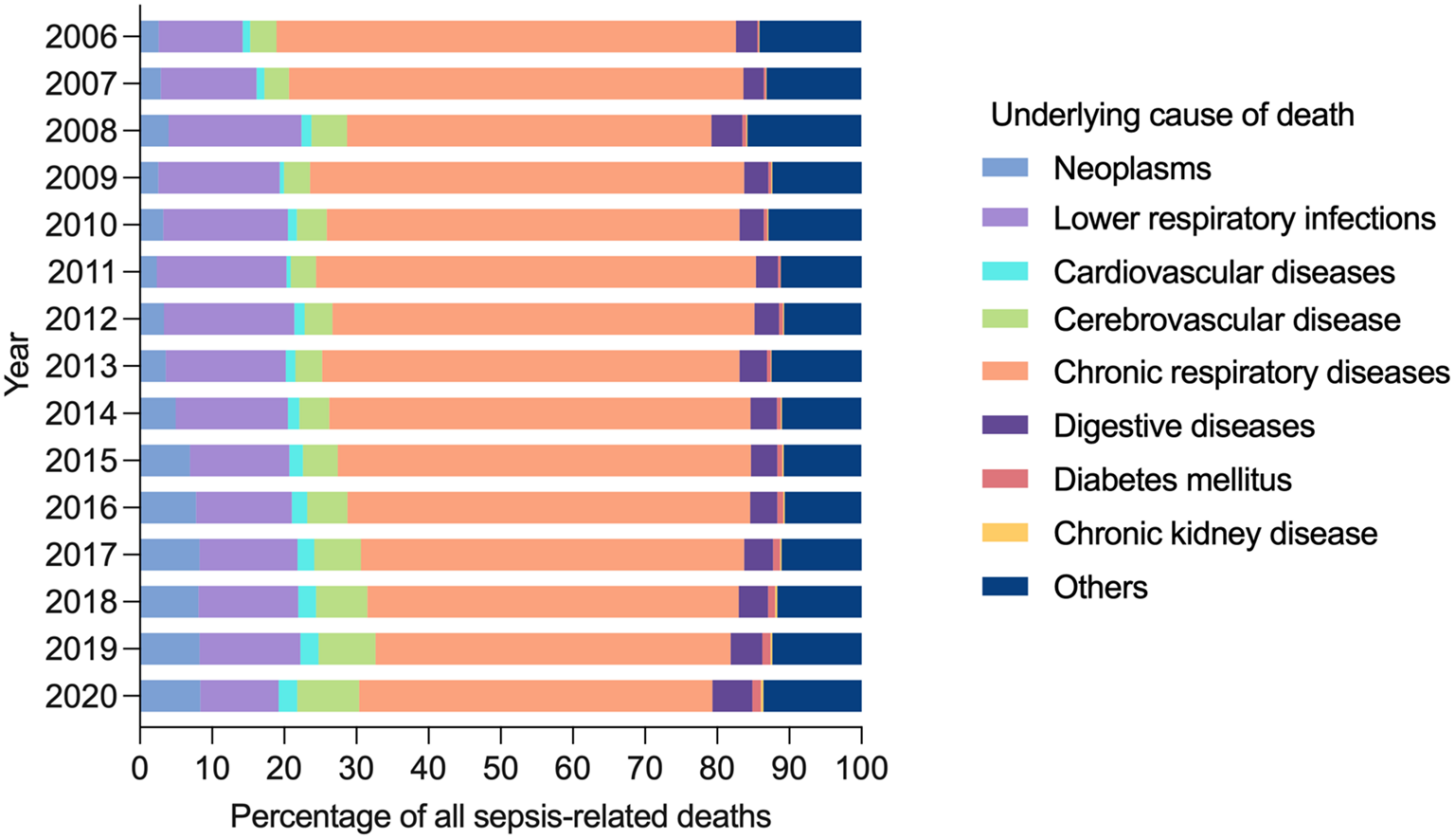
Percentage of all sepsis-related deaths for both sexes in China, 2006–2020, by underlying cause

Chronic respiratory diseases and lower respiratory infections remained the top underlying causes of sepsis-related deaths in 2020. However, rising trends were observed in cerebrovascular disease, neoplasms, digestive diseases, and cardiovascular diseases, which were listed as underlying causes in 2.6–8.6% of sepsis-related deaths in 2020 (Fig. 3). Age-standardized sepsis-related mortality from underlying causes of neoplasms, cardiovascular diseases, cerebrovascular disease, and chronic respiratory diseases were higher than other UCDs. Over the study period, trends of age-standardized mortality from most UCDs remained unchanged, except for declining trends of sepsis-related mortality from chronic respiratory diseases and chronic kidney disease (Additional file 1: Fig. S2).

Nearly, 70% of sepsis-related death occurred at home during the study period, with a decreasing trend from 80.69% in 2006 to 66.95% in 2020 (Additional file 1: Fig. S3). The percentage of sepsis-related death in hospital increased from 15.57% to 30.34%. Substantial variation in the place of sepsis-related death was noted across China. The percentage of septic decedents dying at home in 2020 ranged from 10.6% in Beijing to 87.1% in Jiangsu province, with potentially inverse relation to SDI (*ρ* = − 0.57, *p* < 0.001). The decreasing percentage of sepsis-related death at home was observed in 25 out of 31 provinces, with changes inversely correlated with SDI (*ρ* = − 0.46, *p* = 0.02). (Additional file 1: Fig. S4).

The burden of sepsis-related death varied substantially across the nation (Additional file 1: Table S6, Figs. S5 and S6). In 2020, the highest burden was observed in western China, including Yunnan, Guangxi, Guizhou, Qinghai, Sichuan, and Xinjiang, with age-standardized sepsis-related mortality of 122.8–151.2 per 100,000 population and age-standardized YLLs of 2043.5–2591.3 per 100,000 population. Nonetheless, lower burden was found in northeast and central provinces, including Jilin, Liaoning, Tianjin, Shandong, and Hebei, with age-standardized sepsis-related mortality of 39–45.4 per 100,000 population and age-standardized YLLs of 715.9–798.7 per 100,000 population. Age-standardized mortality decreased or remained stable over the study period in all provinces. The largest decreases were found in Zhejiang (AAPC, − 8.5%; 95%CI − 10.6% to − 6.3%), Hunan (− 7.0%, − 9.1% to − 4.8%), and Ningxia (− 7.0%, − 10.0% to − 4.0%). Trends of age-standardized YLLs were consistent with those of mortality, with the largest decline from 2006 to 2020 observed in Zhejiang (− 66.1%), Ningxia (− 65.3%), and Shanxi (− 61.7%).

**Fig. 4:**
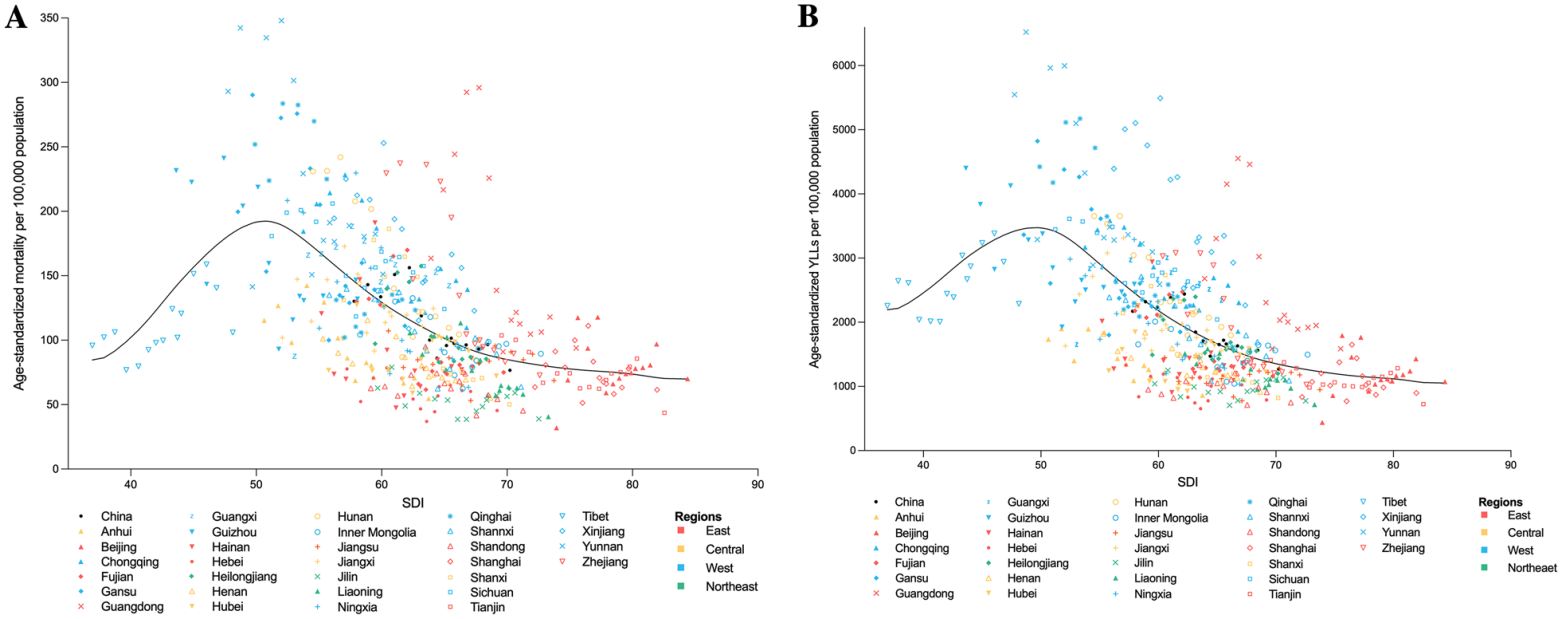
Age-standardized sepsis-related (**A**) mortality and (**B**) YLLs per 100,000 population in China, 2006–2020, by SDI. Black line indicates expected values based on SDI. For each province, points from left to right depict estimates from each year from 2006 to 2020. Each color represents one of the four geographical regions including East (n = 10), Central (n = 6), West (n = 12), and Northeast (n = 3). *SDI* Sociodemographic Index, *YLLs* years of life lost

At national and provincial level, SDI was inversely correlated with age-standardized sepsis-related mortality (*ρ* = − 0.59, *p* < 0.001) and YLLs (*ρ* = − 0.65, *p* < 0.001) (Fig. 4). With a non-linear nature-based solely on SDI, the expected burden of sepsis-related mortality and YLLs declined with increasing SDI and slowed down after SDI reached 70. Paradoxically, increasing trends of sepsis-related mortality and YLLs with rising SDI were observed in Tibet. Large disparities were noted across the nation comparing observed versus expected patterns of sepsis-related deaths. In eastern China, sepsis-related mortality and YLLs were close to expected levels based on SDI. The burden of sepsis-related death remained below expected levels and decreased with rising SDI in most central and northeast provinces. In most parts of western China, sepsis-related mortality and YLLs were higher than expected with closing gaps between observed and expected levels.

## Discussion

Our study is the first to provide nationwide, longitudinal, population-based reports of sepsis-related mortality in China. The use of national vital registry records allowed us to capture both in-hospital deaths and those out of hospital settings, with the latter in the majority according to our findings and previous studies [26–28].

A declining trend of age-standardized sepsis-related mortality was found across the study period from 2006 to 2020 with an annual age-standardized mortality of 76.6 and YLLs of 1271 per 100,000 population in 2020. The results are consistent with a recent analysis of Global Burden of Disease Study which demonstrated decreasing trends of sepsis-related mortality from 1990 to 2017 [2]. A stable trend of sepsis-related mortality was noted in the US from 2005 to 2018 in another study [29]. Nonetheless, substantial disparities between population groups and provinces were noted by all the studies.

Our findings are implicative for efforts to reduce the burden of sepsis in China.

First, sepsis-related health loss poses heavy burden on the healthcare system of China. The age-standardized sepsis-related mortality and YLLs rank only next to deaths caused by neoplasms, cardiovascular diseases, and chronic respiratory diseases [11, 18]. Although progress was made to reduce sepsis-related mortality especially among children, there is still urgent needs for public health measures to improve awareness and control of sepsis.

Second, we found that sepsis-related deaths and crude sepsis-related mortality remained stable despite the decreasing trend of age-standardized mortality. These findings might largely be explained by aging of the population in China, since those aged 65 and over nearly doubled during the study period [17]. Meanwhile, there was a substantial growth of the proportion of decedents aged 75 and over among sepsis-related deaths. Sepsis would probably continue to exert huge burden on the aging population of China for years to come. There is a need for cost-effective actions to improve prevention, identification, and management of sepsis among vulnerable population.

Third, sepsis-related mortality and YLLs were higher among male than female throughout the study period. These findings were consistent with multiple studies evaluating regional or global mortality of sepsis [2, 19–21, 29]. Previous studies also found higher incidence of sepsis among male than female [10, 30]. Since the sex differences were persistent in all age groups, provinces, and underlying causes of death in our study, those factors were unlikely to be main drivers of disparities [11]. Several explanations have been proposed by previous studies, including biological factors, lifestyle behaviors, comorbidities, sources of infection, causative pathogens, and differences in assessment and treatment [31–33]. Further studies are needed to address risk factors underlying sex disparities of sepsis-related death.

Fourth, a potential shift was noted from infectious diseases (e.g., lower respiratory infections) to non-communicable diseases (e.g., neoplasms, cardiovascular diseases, and cerebrovascular disease) for underlying causes of sepsis-related deaths, which is consistent with global and national trends [2, 11, 18]. Public health work could target efforts to prevent and manage sepsis in patient groups with chronic diseases and integrate action plans with existing public health systems of other diseases.

Fifth, home remained the predominant place, where septic patients die, but a shift from home to hospital was noted, especially in provinces with higher sociodemographic level. According to our recent analysis of a 0.5-million Chinese cohort followed up for 8 years, 71.5% of deaths occurred at home, with a large proportion discharged from hospital within 7 days prior to death [26]. The distribution of the place of death might be an interplay of cultural and socioeconomic factors. The deeply rooted Chinese culture prompted families to transfer dying patients back home, which highlights substantial and diverse needs for end-of-life care services at the terminal stage of sepsis in China. Meanwhile, our study noted the potentially inverse relation between sepsis-related death at home and sociodemographic level. Previous studies also revealed that patients with older age, lower socioeconomic status and less coverage of health insurance were more likely to die at home [26, 28, 34, 35]. Endeavors to improve access to healthcare resources among septic patients should be prioritized due to the socioeconomic effect on place of death.

Sixth, significant heterogeneity was found in sepsis-related mortality between provinces. Sepsis-related mortality and YLLs were generally higher in western China, and lower in eastern and central provinces. Decreasing or stable trends of sepsis-related mortality were noted in most parts of China. We investigated the cause of the variability using SDI, a development scale associated with public health outcomes. Consistent with a previous study [2] an inverse relation was noted between SDI and sepsis-related mortality or YLLs. Based on SDI values of provinces, observed levels of sepsis-related health loss could be compared with their expected levels on the spectrum of development to evaluate whether their prevention and management of sepsis is in line with expectation. Not all that simple, non-linear relation was shown on both ends of SDI. Increasing mortality with rising SDI in Tibet might be explained by decreasing rate of underreporting of death [36, 37]. On the other end, patients with complicated or severe illnesses from low or middle SDI provinces seek healthcare in high SDI provinces (e.g., Beijing and Shanghai) [38–40]. An analysis revealed that 0.6 million inpatients, who accounted for 20% of total hospitalizations, travelled across the country to Beijing in 2015 [38]. Although the NMSS did not achieve recognition of cross-regional decedents, the phenomenon might have influence on sepsis-related mortality in high SDI provinces. Given the complexity of provincial differences in sepsis-related mortality, further investigations into the causes and complicating factors are necessary.

Our study has several limitations. First, misclassification of sepsis-related deaths might result from underreporting or misdiagnosis of infection. The underreporting rate of deaths caused by infectious diseases was 11.1% in 2011 [37], and it was reported that 2.73% of causes of death in China were coded inaccurately in 2012 [41]. To address the challenges, multiple measures following the strategies of the World Health Organization have been implemented to promote completeness and accuracy of death registration in the NMSS [15, 16]. Second, potential bias might be introduced by the sample population of the NMSS. However, rigorous procedures, as stated above, were used to ensure the representativeness of the mortality surveillance system [15]. Data from the NMSS have been reliably and extensively used to assess the regional, national, and global burden of disease [11, 18]. Third, the ICD coding algorithm, which was based on infection codes of Angus criteria rather than organ dysfunction codes composing sepsis-3 criteria, might lead to overestimation of sepsis-related deaths compared with implicit or explicit sepsis codes. However, organ dysfunction was remarkably underreported due to limited fields on the death certificate for filling complete list of causes. Most decedents identified by the infection codes would be expected to have experienced organ dysfunction as the mechanism of death. Third, the incidence and case–fatality were not reported due to the lack of epidemiologic data.

## Conclusions

Based on the national mortality surveillance system, we demonstrated a substantial burden of sepsis-related health loss in China. Although decreasing trends of age-standardized mortality and YLLs were noted, there are still significant disparities between population groups and provinces. Clinicians, researchers, and policy makers should align efforts to investigate causes of the disparities, establish policies to close the differences, and implement measures to improve outcomes of sepsis.

### Supplementary Information

**Additional file 1: Table S1**. China standard certificate of death. **Table S2**. International Classification of Diseases 10th (ICD-10) Revision for the identification of infection potentially related to sepsis. **Table S3**. International Classification of Diseases (ICD) codes for underlying causes of sepsis-related deaths. **Table S4**. Number of deaths, age-standardized mortality and YLLs related to sepsis in China, for all ages, both sexes, per year from 2006 to 2020. **Table S5**. Number of deaths and mortality related to sepsis in China, 2006–2020, by age. **Table S6**. Number of deaths, age-standardized mortality and YLLs related to sepsis, 2006–2020, by province of China. **Fig. S1**. Percentage of all sepsis-related deaths for (A) male and (B) female in China, 2006–2020, by age. **Fig. S2**. Age-standardized sepsis-related mortality per 100,000 population in China, by underlying cause of death. **Fig. S3**. Place of death of decedents with sepsis in China, 2006–2020. **Fig. S5**. Age-standardized sepsis-related mortality and YLLs per 100,000 population in China, 2006–2020, by province. **Fig. S6**. Age-standardized sepsis-related mortality and YLLs for each year 2006–2020, by province of China.

## Data Availability

The data sets used and/or analysed during the current study are available from the corresponding author on reasonable request.

## Abbreviations

GBD: Global Burden of Disease
WHO: World Health Organization
LMICs: Low- and middle-income countries
NMSS: National Mortality Surveillance System
SDI: Sociodemographic index
YLLs: Years of life lost
DSPs: Disease surveillance points
UCD: Underlying causes of death
IICD-10: International Classification of Diseases 10th
CI: Confidence interval
AAPC: Average annual percentage changes

## References

[1] Singer M, Deutschman CS, Seymour CW, Shankar-Hari M, Annane D, Bauer M (2016). The third international consensus definitions for sepsis and septic shock (Sepsis-3). JAMA.

[2] Rudd KE, Johnson SC, Agesa KM, Shackelford KA, Tsoi D, Kievlan DR (2020). Global, regional, and national sepsis incidence and mortality, 1990–2017: analysis for the global burden of disease study. Lancet.

[3] World Health Organization (2020). Global report on the epidemiology and burden of sepsis: current evidence, identifying gaps and future directions.

[4] Fleischmann-Struzek C, Mellhammar L, Rose N, Cassini A, Rudd KE, Schlattmann P (2020). Incidence and mortality of hospital- and ICU-treated sepsis: results from an updated and expanded systematic review and meta-analysis. Intensive care med.

[5] Fleischmann C, Andr A, Scherag A, Adhikari NKJ, Hartog CS, Tsaganos T (2016). Assessment of global incidence and mortality of hospital-treated sepsis current estimates and limitations. Am J Respir Crit Care Med.

[6] Xie J, Wang H, Kang Y, Zhou L, Liu Z, Qin B (2020). The epidemiology of sepsis in Chinese ICUs: a national cross-sectional survey. Crit Care Med.

[7] Cheng B, Xie G, Yao SL, Wu X, Guo Q, Gu M (2007). Epidemiology of severe sepsis in critically ill surgical patients in ten university hospitals in China. Crit Care Med.

[8] Zhou J, Qian C, Zhao M, Yu X, Kang Y, Ma X (2014). Epidemiology and outcome of severe sepsis and septic shock in intensive care units in Mainland China. PLoS ONE.

[9] Weng L, Zeng X, Yin P, Wang L, Wang C, Jiang W (2018). Sepsis-related mortality in China: a descriptive analysis. Intensive Care Med.

[10] Weng L, Xu Y, Yin P, Wang Y, Chen Y, Liu W (2023). National incidence and mortality of hospitalized sepsis in China. Crit Care.

[11] Zhou M, Wang H, Zhu J, Chen W, Wang L, Liu S (2016). Cause-specific mortality for 240 causes in China during 1990–2013: a systematic subnational analysis for the Global Burden of Disease Study 2013. Lancet.

[12] Kassebaum NJ, Arora M, Barber RM, Bhutta ZA, Brown J, Carter A (2016). Global, regional, and national disability-adjusted life-years (DALYs) for 315 diseases and injuries and healthy life expectancy (HALE), 1990–2015: a systematic analysis for the Global Burden of Disease Study 2015. Lancet.

[13] Lim SS, Allen K, Bhutta ZA, Dandona L, Forouzanfar MH, Fullman N (2016). Measuring the health-related sustainable development goals in 188 countries: a baseline analysis from the Global Burden of Disease Study 2015. Lancet.

[14] Wang H, Abbas KM, Abbasifard M, Abbasi-Kangevari M, Abbastabar H, Abd-Allah F (2020). Global age-sex-specific fertility, mortality, healthy life expectancy (HALE), and population estimates in 204 countries and territories, 1950–2019: a comprehensive demographic analysis for the Global Burden of Disease Study 2019. Lancet.

[15] Liu S, Wu X, Lopez AD, Wang L, Cai Y, Page A (2016). An integrated national mortality surveillance system for death registration and mortality surveillance. China Bull World Health Organ.

[16] World Health Organization. Improving mortality statistics through civil registration and vital statistics systems: strategies for country and partner support. http://www.who.int/healthinfo/civil_registration/CRVS_MortalityStats_Guidance_Nov2014.pdf. Assessed on 19 Feb 2018.

[17] National Bureau of Statistics of China. https://data.stats.gov.cn/english/index.htm. Assessed on 1 Jun 2022.

[18] Vos T, Lim SS, Abbafati C, Abbas KM, Abbasi M, Abbasifard M (2020). Global burden of 369 diseases and injuries in 204 countries and territories, 1990–2019: a systematic analysis for the Global Burden of Disease Study 2019. Lancet.

[19] Taniguchi LU, Bierrenbach AL, Toscano CM, Schettino GP, Azevedo LC (2014). Sepsis-related deaths in Brazil: an analysis of the national mortality registry from 2002 to 2010. Crit Care.

[20] Fedeli U, Piccinni P, Schievano E, Saugo M, Pellizzer G (2016). Growing burden of sepsis-related mortality in northeastern Italy: a multiple causes of death analysis. BMC Infect Dis.

[21] McPherson D, Griffiths C, Williams M, Baker A, Klodawski E, Jacobson B (2013). Sepsis-associated mortality in England: an analysis of multiple cause of death data from 2001 to 2010. BMJ Open.

[22] Angus DC, Linde-Zwirble WT, Lidicker J, Clermont G, Carcillo J, Pinsky MR (2001). Epidemiology of severe sepsis in the United States: analysis of incidence, outcome, and associated costs of care. Crit Care Med.

[23] National Health Commission of the People’s Republic of China. 2021. 2020 Year book of health in the People’s Republic of China. Beijing;

[24] National Health Commission of the People’s Republic of China. 2001. National Standard Classification and Codes of Diseases. Beijing

[25] Clegg LX, Hankey BF, Tiwari R, Feuer EJ, Edwards BK (2009). Estimating average annual per cent change in trend analysis. Stat Med.

[26] Weng L, Hu Y, Sun Z, Yu C, Guo Y, Pei P (2022). Place of death and phenomenon of going home to die in Chinese adults: a prospective cohort study. Lancet Reg Health West Pac.

[27] Kompanje EJO (2009). Should we discharge comatose patients from intensive care to die in their own bed at home after withdrawal of mechanical ventilation?. Intensive Care Med.

[28] Cai J, Zhao H, Coyte PC (2017). Socioeconomic differences and trends in the place of death among elderly people in China. Int J Environ Res Public Health.

[29] Prest J, Sathananthan M, Jeganathan N (2021). Current trends in sepsis-related mortality in the United States. Crit Care Med.

[30] Martin GS, Mannino DM, Eaton S, Moss M (2003). The epidemiology of sepsis in the United States from 1979 through 2000. N Engl J Med.

[31] Esper AM, Moss M, Lewis CA, Nisbet R, Mannino DM, Martin GS (2006). The role of infection and comorbidity: factors that influence disparities in sepsis. Crit Care Med.

[32] Lakbar I, Einav S, Lalevée N, Martin-Loeches I, Pastene B, Leone M (2023). Interactions between gender and sepsis—implications for the future. Microorganisms.

[33] Weng L, Fan J, Yu C, Guo Y, Bian Z, Wei Y (2020). Body-mass index and long-term risk of sepsis-related mortality: a population-based cohort study of 0.5 million Chinese adults. Crit Care.

[34] Li Z, Jiang S, Xu C, Lu F, He R, Pan Z (2019). Determinants of place of death for end-stage cancer patients: evidence from China. Int J Qual Heal Care.

[35] Wang W, Liu Y, Ye P, Liu J, Yin P, Qi J (2022). Trends and associated factors in place of death among individuals with cardiovascular disease in China, 2008–2020: a population-based study. Lancet Reg Health West Pac.

[36] Zeng X, Adair T, Wang L, Yin P, Qi J, Liu Y (2020). Measuring the completeness of death registration in 2844 Chinese counties in 2018. BMC Med.

[37] Guo K, Yin P, Wang L, Ji Y, Li Q, Bishai D (2015). Propensity score weighting for addressing under-reporting in mortality surveillance: A proof-of-concept study using the nationally representative mortality data in China. Popul Health Metr.

[38] Yang Y, Wang Y (2022). Analysis of the characteristics of cross-regional patient groups and differences in hospital service utilization in Beijing. Int J Environ Res Public Health.

[39] Li L, Zhou Q, Yin T, Ji Z, Zhang L (2021). Does the direct settlement policy of trans-provincial outpatient expenses aggravate the siphoning effect? An empirical study on yangtze river delta, China. Int J Environ Res Public Health.

[40] National Health Commission of the People’s Republic of China. National Report on the Service, Quality and Safety in Medical Care System. Beijing;2020.

[41] Chinese Center for Disease Control and Prevention. Report of cause-of-death surveillance in China 2012. Beijing;2013.

