# Supplementary material for: Temporal trends of sepsis-related mortality in China, 2006–2020: a population-based study
Source: Ann Intensive Care. 2023 Aug 14;13:71. doi: 10.1186/s13613-023-01166-1 (PMC10425320; doi:10.1186/s13613-023-01166-1)
Supplement: Supplementary file 1 — Additional file 1: Table S1. China standard certificate of death. Table S2. International Classification of Diseases 10th (ICD-10) Revision for the identification of infection potentially related to sepsis. Table S3. International Classification of Diseases (ICD) codes for underlying causes of sepsis-related deaths. Table S4. Number of deaths, age-standardized mortality and YLLs related to sepsis in China, for all ages, both sexes, per year from 2006 to 2020. Table S5. Number of deaths and mortality related to sepsis in China, 2006–2020, by age. Table S6. Number of deaths, age-standardized mortality and YLLs related to sepsis, 2006–2020, by province of China. Fig. S1. Percentage of all sepsis-related deaths for (A) male and (B) female in China, 2006–2020, by age. Fig. S2. Age-standardized sepsis-related mortality per 100,000 population in China, by underlying cause of death. Fig. S3. Place of death of decedents with sepsis in China, 2006–2020. Fig. S5. Age-standardized sepsis-related mortality and YLLs per 100,000 population in China, 2006–2020, by province. Fig. S6. Age-standardized sepsis-related mortality and YLLs for each year 2006–2020, by province of China. [file 13613_2023_1166_MOESM1_ESM.docx]

**Appendix to**

**Temporal trends of sepsis-related mortality in China, 2006-2020: A population-based study**

[Table S1. China standard certificate of death (translated edition) 1](#_Toc138033685)

[Table S2. International Classification of Diseases 10th (ICD-10) Revision for the identification of infection potentially related to sepsis 2](#_Toc138033686)

[Table S3. International Classification of Diseases (ICD) codes for underlying causes of sepsis-related deaths. 19](#_Toc138033687)

[Table S4. Number of deaths, age-standardized mortality and YLLs related to sepsis in China, for all ages, both sexes, per year from 2006 to 2020. 20](#_Toc138033688)

[Table S5. Number of deaths and mortality related to sepsis in China, 2006-2020, by age. 21](#_Toc138033689)

[Table S6. Number of deaths, age-standardized mortality and YLLs related to sepsis, 2006-2020, by province of China. 22](#_Toc138033690)

[Fig. S1. Percentage of all sepsis-related deaths for (A) male and (B) female in China, 2006-2020, by age. 24](#_Toc138033691)

[Fig. S2. Age-standardized sepsis-related mortality per 100,000 population in China, by underlying cause of death. 24](#_Toc138033692)

[Fig. S3. Place of death of decedents with sepsis in China, 2006-2020. 25](#_Toc138033693)

[Fig. S5. Age-standardized sepsis-related mortality and YLLs per 100,000 population in China, 2006-2020, by province. 26](#_Toc138033694)

[Fig. S6. Age-standardized sepsis-related mortality and YLLs for each year 2006-2020, by province of China. 27](#_Toc138033695)

# Table S1. China standard certificate of death (translated edition)

| **CHINA STANDARD CERTIFICATE OF DEATH (TRANSLATED BY INVESTIGATORS)** | | | | | | | | | | |
| --- | --- | --- | --- | --- | --- | --- | --- | --- | --- | --- |
| NAME |  | SEX | |  | ETHNICITY |  | | | NATION |  |
| CITIZEN ID TYPE |  | CITIZEN ID NUMBER | |  | AGE |  | | | MARITAL STATUS |  |
| DATE OF BIRTH |  | EDUCATIONAL STATUS | |  | OCCUPATION |  | | | | |
| DATE OF DEATH |  | PLACE OF DEATH | | 1, Hospital ; 2, Prehospital; 3, Home; 4, Nursing home; 9,Other Places; 0, NA | | | PREGENANCY STATUS | | |  |
| WORKING PLACE |  | REGISTRATION ADDRESS | |  | | | MAILING ADDRESS | |  | |
| NAMES OF RELATIVES |  | TEL | |  | | | ADDRESS OF RELATIVES | |  | |
|  | | | CAUSE OF DEATH (ICD CODE) | | | | | | Approximate interval: Onset to death | |
| I. (a) IMMEDIATE CAUSE | | |  | | | | | |  | |
| (b) as a cause of (a) | | |  | | | | | |  | |
| (c) as a cause of (b) | | |  | | | | | |  | |
| (d) as a cause of (c) | | |  | | | | | |  | |
| II. UNDERLYING CAUSE (not directly related death) | | |  | | | | | |  | |
| HEALTH CARE INSTITUTIONS THAT DETERMINED THE CAUSES OF DEATHS | 1, Tertiary care; 2, Secondary care; 3, Community care; 4, Primary care in village; 9, Other health care institution; 0, Home care | | | | | | STRONGEST EVIDENCE OF DIAGNOSIS | | 1, Autopsy;2, Pathology; 3, Surgery;4, Bedside clinical plus laboratory findings;5, Bedside clinical findings;5, Speculation;9, No available | |
| SIGNATURE OF PHYSICIAN |  | HEALTH CARE INSTITUTION | | | | | DATE SIGNED | | | |
| UNDERLING DISEASE OF DEATH (FILLED BY CDC STAFF) | | | | | | | | ICD code (FILLED BY CDC STAFF) | | |

# Table S2. International Classification of Diseases 10th (ICD-10) Revision for the identification of infection potentially related to sepsis

| **ICD code** | **Infection description** |
| --- | --- |
| A00 | Cholera |
| A00.0 | Cholera due to Vibrio cholerae 01, biovar cholerae |
| A00.1 | Cholera due to Vibrio cholerae 01, biovar eltor |
| A00.9 | Cholera, unspecified |
| A01 | Typhoid and paratyphoid fevers |
| A01.0 | Typhoid fever |
| A01.1 | Paratyphoid fever A |
| A01.2 | Paratyphoid fever B |
| A01.3 | Paratyphoid fever C |
| A01.4 | Paratyphoid fever, unspecified |
| A02 | Other salmonella infections |
| A02.0 | Salmonella enteritis |
| A02.1 | Salmonella septicemia |
| A02.2 | Localized salmonella infections |
| A02.8 | Other specified salmonella infections |
| A02.9 | Salmonella infection, unspecified |
| A03 | Shigellosis |
| A03.0 | Shigellosis due to Shigella dysenteriae |
| A03.1 | Shigellosis due to Shigella flexneri |
| A03.2 | Shigellosis due to Shigella boydii |
| A03.3 | Shigellosis due to Shigella sonnei |
| A03.9 | Shigellosis, unspecified |
| A04 | Other bacterial intestinal infections |
| A04.0 | Enteropathogenic Escherichia coli infection |
| A04.2 | Enteroinvasive Escherichia coli infection |
| A04.3 | Enterohemorrhagic Escherichia coli infection |
| A04.4 | Other intestinal Escherichia coli infections |
| A04.5 | Campylobacter enteritis |
| A04.6 | Enteritis due to Yersinia enterocolitica |
| A04.7 | Enterocolitis due to Clostridium difficile |
| A04.8 | Other specified bacterial intestinal infections |
| A04.9 | Bacterial intestinal infection, unspecified |
| A05 | Other bacterial foodborne intoxications, not elsewhere classified |
| A05.0 | Foodborne staphylococcal intoxication |
| A05.1 | Botulism |
| A05.2 | Foodborne Clostridium perfringens [Clostridium welchii] intoxication |
| A05.3 | Foodborne Vibrio parahemolyticus intoxication |
| A05.4 | Foodborne Bacillus cereus intoxication |
| A05.8 | Other specified bacterial foodborne intoxications |
| A05.9 | Bacterial foodborne intoxication, unspecified |
| A06 | Amebiasis |
| A06.0 | Acute amebic dysentery |
| A06.2 | Amebic nondysenteric colitis |
| A06.4 | Amebic liver abscess |
| A06.5 | Amebic lung abscess |
| A06.6 | Amebic brain abscess |
| A06.7 | Cutaneous amebiasis |
| A08 | Viral and other specified intestinal infections |
| A08.1 | Acute gastroenteropathy due to Norwalk agent |
| A08.2 | Adenoviral enteritis |
| A08.3 | Other viral enteritis |
| A08.4 | Viral intestinal infection, unspecified |
| A08.5 | Other specified intestinal infections |
| A09 | Diarrhea and gastroenteritis of infectious origin |
| A09.0 | Other and unspecified gastroenteritis and colitis of infectious origin |
| A15 | Respiratory tuberculosis, bacteriologically and histologically confirmed |
| A15.0 | Tuberculosis of lung, confirmed by sputum microscopy with or without culture |
| A15.1 | Tuberculosis of lung, confirmed by culture only |
| A15.2 | Tuberculosis of lung, confirmed histologically |
| A15.3 | Tuberculosis of lung, confirmed by unspecified means |
| A15.5 | Tuberculosis of larynx, trachea and bronchus, confirmed bacteriologically and histologically |
| A15.6 | Tuberculous pleurisy, confirmed bacteriologically and histologically |
| A15.7 | Primary respiratory tuberculosis, confirmed bacteriologically and histologically |
| A15.8 | Other respiratory tuberculosis, confirmed bacteriologically and histologically |
| A15.9 | Respiratory tuberculosis unspecified, confirmed bacteriologically and histologically |
| A16 | Respiratory tuberculosis, not confirmed bacteriologically or histologically |
| A16.0 | Tuberculosis of lung, bacteriologically and histologically negative |
| A16.1 | Tuberculosis of lung, bacteriological and histological examination not done |
| A16.2 | Tuberculosis of lung, without mention of bacteriological or histological confirmation |
| A16.4 | Tuberculosis of larynx, trachea, and bronchus, without mention of bacteriological or histological confirmation |
| A16.5 | Tuberculous pleurisy, without mention of bacteriological or histological confirmation |
| A16.7 | Primary respiratory tuberculosis without mention of bacteriological or histological confirmation |
| A16.8 | Other respiratory tuberculosis, without mention of bacteriological or histological confirmation |
| A16.9 | Respiratory tuberculosis unspecified, without mention of bacteriological or histological confirmation |
| A19 | Miliary tuberculosis |
| A19.0 | Acute miliary tuberculosis of a single specified site |
| A19.2 | Acute miliary tuberculosis, unspecified |
| A19.8 | Other miliary tuberculosis |
| A19.9 | Miliary tuberculosis, unspecified |
| A20 | Plague |
| A20.0 | Bubonic plague |
| A20.1 | Cellulocutaneous plague |
| A20.2 | Pneumonic plague |
| A20.3 | Plague meningitis |
| A20.7 | Septicemic plague |
| A20.8 | Other forms of plague |
| A20.9 | Plague, unspecified |
| A21 | Tularemia |
| A21.0 | Ulceroglandular tularemia |
| A21.1 | Oculoglandular tularemia |
| A21.2 | Pulmonary tularemia |
| A21.3 | Gastrointestinal tularemia |
| A21.7 | Generalized tularemia |
| A21.8 | Other forms of tularemia |
| A21.9 | Tularemia, unspecified |
| A22 | Anthrax |
| A22.1 | Pulmonary anthrax |
| A22.2 | Gastrointestinal anthrax |
| A22.7 | Anthrax septicemia |
| A22.8 | Other forms of anthrax |
| A22.9 | Anthrax, unspecified |
| A23 | Brucellosis |
| A23.0 | Brucellosis due to Brucella melitensis |
| A23.1 | Brucellosis due to Brucella abortus |
| A23.2 | Brucellosis due to Brucella suis |
| A23.3 | Brucellosis due to Brucella canis |
| A23.8 | Other brucellosis |
| A23.9 | Brucellosis, unspecified |
| A24 | Glanders and melioidosis |
| A24.0 | Glanders |
| A24.1 | Acute and fulminating melioidosis |
| A24.3 | Other melioidosis |
| A24.4 | Melioidosis, unspecified |
| A25 | Rat-bite fevers |
| A25.0 | Spirillosis |
| A25.1 | Streptobacillosis |
| A25.9 | Rat-bite fever, unspecified |
| A26 | Erysipeloid |
| A26.0 | Cutaneous erysipeloid |
| A26.7 | Erysipelothrix septicemia |
| A26.8 | Other forms of erysipeloid |
| A26.9 | Erysipeloid, unspecified |
| A27 | Leptospirosis |
| A27.0 | Leptospirosis icterohemorrhagica |
| A27.8 | Other forms of leptospirosis |
| A27.9 | Leptospirosis, unspecified |
| A28 | Other zoonotic bacterial diseases, not elsewhere classified |
| A28.0 | Pasteurellosis |
| A28.1 | Cat-scratch disease |
| A28.2 | Extraintestinal yersiniosis |
| A28.8 | Other specified zoonotic bacterial diseases, not elsewhere classified |
| A28.9 | Zoonotic bacterial disease, unspecified |
| A31 | Infection due to other mycobacteria |
| A31.0 | Pulmonary mycobacterial infection |
| A31.1 | Cutaneous mycobacterial infection |
| A31.8 | Other mycobacterial infections |
| A31.9 | Mycobacterial infection, unspecified |
| A32 | Listeriosis |
| A32.0 | Cutaneous listeriosis |
| A32.1 | Listerial meningitis and meningoencephalitis |
| A32.7 | Listerial septicemia |
| A32.8 | Other forms of listeriosis |
| A32.9 | Listeriosis, unspecified |
| A33 | Tetanus neonatorum |
| A34 | Obstetrical tetanus |
| A35 | Other tetanus |
| A36 | Diphtheria |
| A36.0 | Pharyngeal diphtheria |
| A36.1 | Nasopharyngeal diphtheria |
| A36.2 | Laryngeal diphtheria |
| A36.3 | Cutaneous diphtheria |
| A36.8 | Other diphtheria |
| A36.9 | Diphtheria, unspecified |
| A37 | Whooping cough |
| A37.0 | Whooping cough due to Bordetella pertussis |
| A37.1 | Whooping cough due to Bordetella parapertussis |
| A37.8 | Whooping cough due to other Bordetella species |
| A37.9 | Whooping cough, unspecified |
| A38 | Scarlet fever |
| A39 | Meningococcal infection |
| A39.0 | Meningococcal meningitis |
| A39.1 | Waterhouse-Friderichsen syndrome |
| A39.2 | Acute meningococcemia |
| A39.4 | Meningococcemia, unspecified |
| A39.5 | Meningococcal heart disease |
| A39.8 | Other meningococcal infections |
| A39.9 | Meningococcal infection, unspecified |
| A40 | Streptococcal septicemia |
| A40.0 | Septicemia due to streptococcus, group A |
| A40.1 | Septicemia due to streptococcus, group B |
| A40.2 | Septicemia due to streptococcus, group D |
| A40.3 | Septicemia due to Streptococcus pneumoniae |
| A40.8 | Other streptococcal septicemia |
| A40.9 | Streptococcal septicemia, unspecified |
| A41 | Other septicemia |
| A41.0 | Septicemia due to Staphylococcus aureus |
| A41.1 | Septicemia due to other specified staphylococcus |
| A41.2 | Septicemia due to unspecified staphylococcus |
| A41.3 | Septicemia due to Hemophilus influenzae |
| A41.4 | Septicemia due to anaerobes |
| A41.5 | Septicemia due to other Gram<U+2011>negative organisms |
| A41.8 | Other specified septicemia |
| A41.9 | Septicemia, unspecified |
| A42 | Actinomycosis |
| A42.0 | Pulmonary actinomycosis |
| A42.1 | Abdominal actinomycosis |
| A42.2 | Cervicofacial actinomycosis |
| A42.7 | Actinomycotic septicemia |
| A42.8 | Other forms of actinomycosis |
| A42.9 | Actinomycosis, unspecified |
| A43 | Nocardiosis |
| A43.0 | Pulmonary nocardiosis |
| A43.1 | Cutaneous nocardiosis |
| A43.8 | Other forms of nocardiosis |
| A43.9 | Nocardiosis, unspecified |
| A46 | Erysipelas |
| A48 | Other bacterial diseases, not elsewhere classified |
| A48.0 | Gas gangrene |
| A48.1 | Legionnaires' disease |
| A48.2 | Nonpneumonic Legionnaires' disease [Pontiac fever] |
| A48.3 | Toxic shock syndrome |
| A48.8 | Other specified bacterial diseases |
| A49 | Bacterial infection of unspecified site |
| A49.0 | Staphylococcal infection, unspecified |
| A49.1 | Streptococcal infection, unspecified |
| A49.2 | Hemophilus influenzae infection, unspecified |
| A49.3 | Mycoplasma infection, unspecified |
| A49.8 | Other bacterial infections of unspecified site |
| A49.9 | Bacterial infection, unspecified |
| A50 | Congenital syphilis |
| A50.0 | Early congenital syphilis, symptomatic |
| A50.1 | Early congenital syphilis, latent |
| A50.2 | Early congenital syphilis, unspecified |
| A50.3 | Late congenital syphilitic oculopathy |
| A50.4 | Late congenital neurosyphilis [juvenile neurosyphilis] |
| A50.5 | Other late congenital syphilis, symptomatic |
| A50.6 | Late congenital syphilis, latent |
| A50.7 | Late congenital syphilis, unspecified |
| A50.9 | Congenital syphilis, unspecified |
| A51 | Early syphilis |
| A51.0 | Primary genital syphilis |
| A51.1 | Primary anal syphilis |
| A51.2 | Primary syphilis of other sites |
| A51.3 | Secondary syphilis of skin and mucous membranes |
| A51.4 | Other secondary syphilis |
| A51.5 | Early syphilis, latent |
| A51.9 | Early syphilis, unspecified |
| A53 | Other and unspecified syphilis |
| A53.0 | Latent syphilis, unspecified as early or late |
| A53.9 | Syphilis, unspecified |
| A54 | Gonococcal infection |
| A54.0 | Gonococcal infection of lower genitourinary tract without periurethral or accessory gland abscess |
| A54.1 | Gonococcal infection of lower genitourinary tract with periurethral and accessory gland abscess |
| A54.2 | Gonococcal pelviperitonitis and other gonococcal genitourinary infections |
| A54.3 | Gonococcal infection of eye |
| A54.4 | Gonococcal infection of musculoskeletal system |
| A54.5 | Gonococcal pharyngitis |
| A54.6 | Gonococcal infection of anus and rectum |
| A54.8 | Other gonococcal infections |
| A54.9 | Gonococcal infection, unspecified |
| A55 | Chlamydial lymphogranuloma (venereum) |
| A56.0 | Chlamydial infection of lower genitourinary tract |
| A56.1 | Chlamydial infection of pelviperitoneum and other genitourinary organs |
| A56.2 | Chlamydial infection of genitourinary tract, unspecified |
| A56.3 | Chlamydial infection of anus and rectum |
| A60 | Anogenital herpesviral [herpes simplex] infection |
| A60.0 | Herpesviral infection of genitalia and urogenital tract |
| A60.1 | Herpesviral infection of perianal skin and rectum |
| A69 | Other spirochetal infections |
| A69.0 | Necrotizing ulcerative stomatitis |
| A69.1 | Other Vincent's infections |
| A69.8 | Other specified spirochetal infections |
| A69.9 | Spirochetal infection, unspecified |
| A71 | Trachoma |
| A71.0 | Initial stage of trachoma |
| A71.1 | Active stage of trachoma |
| A71.9 | Trachoma, unspecified |
| A74.9 | Chlamydial infection, unspecified |
| A75 | Typhus fever |
| A75.0 | Epidemic louse-borne typhus fever due to Rickettsia prowazekii |
| A75.1 | Recrudescent typhus [Brill's disease] |
| A75.2 | Typhus fever due to Rickettsia typhi |
| A75.3 | Typhus fever due to Rickettsia tsutsugamushi |
| A75.9 | Typhus fever, unspecified |
| A77.8 | Other spotted fevers |
| A79 | Other rickettsioses |
| A79.0 | Trench fever |
| A79.1 | Rickettsialpox due to Rickettsia akari |
| A79.8 | Other specified rickettsioses |
| A79.9 | Rickettsiosis, unspecified |
| A81 | Atypical virus infections of central nervous system |
| A81.0 | Creutzfeldt-Jakob disease |
| A81.1 | Subacute sclerosing panencephalitis |
| A81.2 | Progressive multifocal leukoencephalopathy |
| A81.8 | Other atypical virus infections of central nervous system |
| A81.9 | Atypical virus infection of central nervous system, unspecified |
| A82 | Rabies |
| A82.0 | Sylvatic rabies |
| A82.1 | Urban rabies |
| A82.9 | Rabies, unspecified |
| A83 | Mosquito-borne viral encephalitis |
| A83.0 | Japanese encephalitis |
| A83.1 | Western equine encephalitis |
| A83.2 | Eastern equine encephalitis |
| A83.3 | St. Louis encephalitis |
| A83.4 | Australian encephalitis |
| A83.5 | California encephalitis |
| A83.6 | Rocio virus disease |
| A83.8 | Other mosquito-borne viral encephalitis |
| A83.9 | Mosquito-borne viral encephalitis, unspecified |
| A84 | Tick-borne viral encephalitis |
| A84.0 | Far Eastern tick-borne encephalitis [Russian spring-summer encephalitis] |
| A84.1 | Central European tick-borne encephalitis |
| A84.8 | Other tick-borne viral encephalitis |
| A84.9 | Tick-borne viral encephalitis, unspecified |
| A85 | Other viral encephalitis, not elsewhere classified |
| A85.0 | Enteroviral encephalitis |
| A85.1 | Adenoviral encephalitis |
| A85.2 | Arthropod-borne viral encephalitis, unspecified |
| A85.8 | Other specified viral encephalitis |
| A86 | Unspecified viral encephalitis |
| A87 | Viral meningitis |
| A87.0 | Enteroviral meningitis |
| A87.1 | Adenoviral meningitis |
| A87.2 | Lymphocytic choriomeningitis |
| A87.8 | Other viral meningitis |
| A87.9 | Viral meningitis, unspecified |
| A88 | Other viral infections of the central nervous system, not elsewhere classified |
| A88.0 | Enteroviral exanthematous fever [Boston exanthem] |
| A88.8 | Other specified viral infections of central nervous system |
| A89 | Unspecified viral infection of central nervous system |
| A92 | Other mosquito-borne viral fevers |
| A92.0 | Chikungunya virus disease |
| A92.1 | O'nyong-nyong fever |
| A92.2 | Venezuelan equine fever |
| A92.3 | West Nile fever |
| A92.4 | Rift Valley fever |
| A92.8 | Other specified mosquito-borne viral fevers |
| A92.9 | Mosquito-borne viral fever, unspecified |
| A93 | Other arthropod-borne viral fevers, not elsewhere classified |
| A93.0 | Oropouche virus disease |
| A93.1 | Sandfly fever |
| A93.2 | Colorado tick fever |
| A93.8 | Other specified arthropod-borne viral fevers |
| A94 | Unspecified arthropod-borne viral fever |
| A96.8 | Other arenaviral hemorrhagic fevers |
| A98 | Other viral hemorrhagic fevers, not elsewhere classified |
| A98.5 | Hemorrhagic fever with renal syndrome |
| A99 | Unspecified viral hemorrhagic fever |
| B00 | Herpesviral [herpes simplex] infections |
| B00.1 | Herpesviral vesicular dermatitis |
| B00.2 | Herpesviral gingivostomatitis and pharyngotonsillitis |
| B00.3 | Herpesviral meningitis |
| B00.4 | Herpesviral encephalitis |
| B00.7 | Disseminated herpesviral disease |
| B00.8 | Other forms of herpesviral infection |
| B00.9 | Herpesviral infection, unspecified |
| B01 | Varicella [chickenpox] |
| B01.0 | Varicella meningitis |
| B01.2 | Varicella pneumonia |
| B01.8 | Varicella with other complications |
| B01.9 | Varicella without complications |
| B02 | Zoster [herpes zoster] |
| B02.0 | Zoster encephalitis |
| B02.1 | Zoster meningitis |
| B02.2 | Zoster with other nervous system involvement |
| B02.3 | Zoster ocular disease |
| B02.7 | Disseminated zoster |
| B02.8 | Zoster with other complications |
| B02.9 | Zoster without complication |
| B05 | Measles |
| B05.0 | Measles complicated by encephalitis |
| B05.1 | Measles complicated by meningitis |
| B05.2 | Measles complicated by pneumonia |
| B05.8 | Measles with other complications |
| B06 | Rubella [German measles] |
| B06.0 | Rubella with neurological complications |
| B08 | Other viral infections characterized by skin and mucous membrane lesions, not elsewhere classified |
| B08.8 | Other specified viral infections characterized by skin and mucous membrane lesions |
| B09 | Unspecified viral infection characterized by skin and mucous membrane lesions |
| B20 | Human immunodeficiency virus [HIV] disease with infectious and parasitic diseases |
| B20.0 | HIV disease with mycobacterial infection |
| B20.1 | HIV disease with other bacterial infections |
| B20.2 | HIV disease with cytomegaloviral disease |
| B20.3 | HIV disease with other viral infections |
| B20.4 | HIV disease with candidiasis |
| B20.5 | HIV disease with other mycoses |
| B20.6 | HIV disease with Pneumocystis carinii pneumonia |
| B20.7 | HIV disease with multiple infections |
| B20.8 | HIV disease with other infectious and parasitic diseases |
| B20.9 | HIV disease with unspecified infectious or parasitic disease |
| B25.0 | Cytomegaloviral pneumonitis |
| B25.1 | Cytomegaloviral hepatitis |
| B25.2 | Cytomegaloviral pancreatitis |
| B25.9 | Cytomegaloviral disease, unspecified |
| B26.1 | Mumps meningitis |
| B26.2 | Mumps encephalitis |
| B26.3 | Mumps pancreatitis |
| B26.8 | Mumps with other complications |
| B26.9 | Mumps without complication |
| B27.1 | Cytomegaloviral mononucleosis |
| B27.9 | Infectious mononucleosis, unspecified |
| B30.2 | Viral pharyngoconjunctivitis |
| B33.2 | Viral carditis |
| B33.4 | Hantavirus (cardio)-pulmonary syndrome [HPS][HCPS] |
| B34 | Viral infection of unspecified site |
| B34.1 | Enterovirus infection, unspecified |
| B34.8 | Other viral infections of unspecified site |
| B34.9 | Viral infection, unspecified |
| B35.0 | Tinea barbae and tinea capitis |
| B37.0 | Candidal stomatitis |
| B37.1 | Pulmonary candidiasis |
| B37.4 | Candidiasis of other urogenital sites |
| B37.6 | Candidal endocarditis |
| B37.7 | Candidal septicemia |
| B37.8 | Candidiasis of other sites |
| B38.0 | Acute pulmonary coccidioidomycosis |
| B38.2 | Pulmonary coccidioidomycosis, unspecified |
| B38.4 | Coccidioidomycosis meningitis |
| B39 | Histoplasmosis |
| B41.0 | Pulmonary paracoccidioidomycosis |
| B42.0 | Pulmonary sporotrichosis |
| B43.1 | Pheomycotic brain abscess |
| B43.2 | Subcutaneous pheomycotic abscess and cyst |
| B44.0 | Invasive pulmonary aspergillosis |
| B44.1 | Other pulmonary aspergillosis |
| B45 | Cryptococcosis |
| B45.1 | Cerebral cryptococcosis |
| B45.7 | Disseminated cryptococcosis |
| B46.0 | Pulmonary mucormycosis |
| B46.8 | Other zygomycoses |
| B48 | Other mycoses, not elsewhere classified |
| B48.2 | Allescheriasis |
| B48.4 | Penicillosis |
| B48.7 | Opportunistic mycoses |
| B48.8 | Other specified mycoses |
| B49 | Unspecified mycosis |
| B50 | Plasmodium falciparum malaria |
| B50.0 | Plasmodium falciparum malaria with cerebral complications |
| B50.9 | Plasmodium falciparum malaria, unspecified |
| B51 | Plasmodium vivax malaria |
| B51.0 | Plasmodium vivax malaria with rupture of spleen |
| B53.8 | Other parasitologically confirmed malaria, not elsewhere classified |
| B54 | Unspecified malaria |
| B55.0 | Visceral leishmaniasis |
| B57.0 | Acute Chagas' disease with heart involvement |
| B58.2 | Toxoplasma meningoencephalitis |
| B59 | Pneumocystosis |
| B65 | Schistosomiasis [bilharziasis] |
| B65.1 | Schistosomiasis due to Schistosoma mansoni [intestinal schistosomiasis] |
| B65.8 | Other schistosomiases |
| B66 | Other fluke infections |
| B66.1 | Clonorchiasis |
| B66.8 | Other specified fluke infections |
| B67.0 | Echinococcus granulosus infection of liver |
| B67.3 | Echinococcus granulosus infection, other and multiple sites |
| B67.8 | Echinococcosis, unspecified, of liver |
| B67.9 | Echinococcosis, other and unspecified |
| B68.1 | Tenia saginata teniasis |
| B69.0 | Cysticercosis of central nervous system |
| B69.1 | Cysticercosis of eye |
| B69.8 | Cysticercosis of other sites |
| B69.9 | Cysticercosis, unspecified |
| B74.9 | Filariasis, unspecified |
| B76 | Hookworm diseases |
| B76.9 | Hookworm disease, unspecified |
| B77.0 | Ascariasis with intestinal complications |
| B77.8 | Ascariasis with other complications |
| B77.9 | Ascariasis, unspecified |
| B88.8 | Other specified infestations |
| B89 | Unspecified parasitic disease |
| B95 | Streptococcus and staphylococcus as the cause of diseases classified to other chapters |
| B95.3 | Streptococcus pneumoniae as the cause of diseases classified to other chapters |
| B95.4 | Other streptococcus as the cause of diseases classified to other chapters |
| B95.5 | Unspecified streptococcus as the cause of diseases classified to other chapters |
| B95.6 | Staphylococcus aureus as the cause of diseases classified to other chapters |
| B95.7 | Other staphylococcus as the cause of diseases classified to other chapters |
| B95.8 | Unspecified staphylococcus as the cause of diseases classified to other chapters |
| B96.0 | Mycoplasma pneumoniae [M. pneumoniae] as the cause of diseases classified to other chapters |
| B96.1 | Klebsiella pneumoniae [K. pneumoniae] as the cause of diseases classified to other chapters |
| B96.2 | Escherichia coli [E. coli] as the cause of diseases classified to other chapters |
| B96.4 | Proteus (mirabilis)(morganii) as the cause of diseases classified to other chapters |
| B96.5 | Pseudomonas (aeruginosa) as the cause of diseases classified to other chapters |
| B96.8 | Other specified bacterial agents as the cause of diseases classified to other chapters |
| B97.4 | Respiratory syncytial virus as the cause of diseases classified to other chapters |
| B97.7 | Papillomavirus as the cause of diseases classified to other chapters |
| B97.8 | Other viral agents as the cause of diseases classified to other chapters |
| B99 | Other and unspecified infectious diseases |
| B99 | Other infectious diseases |
| D73.3 | Abscess of spleen |
| D76.2 | Haemophagocytic syndrome, infection-associated |
| G00 | Bacterial meningitis, not elsewhere classified |
| G00.0 | Hemophilus meningitis |
| G00.1 | Pneumococcal meningitis |
| G00.2 | Streptococcal meningitis |
| G00.3 | Staphylococcal meningitis |
| G00.8 | Other bacterial meningitis |
| G00.9 | Bacterial meningitis, unspecified |
| G01 | Meningitis in bacterial disease classified elsewhere |
| G04 | Encephalitis, myelitis and encephalomyelitis |
| G05 | Encephalitis, myelitis and encephalomyelitis in diseases classified elsewhere |
| G06 | Intracranial and intraspinal abscess and granuloma |
| G07 | Intracranial and intraspinal abscess and granuloma in diseases classified elsewhere |
| G08 | Intracranial and intraspinal phlebitis and thrombophlebitis |
| G94.0 | Hydrocephalus in infectious and parasitic diseases classified elsewhere |
| H13.1 | Conjunctivitis in infectious and parasitic diseases classified elsewhere |
| H44.0 | Purulent endophthalmitis |
| H61.0 | Perichondritis of external ear |
| H62.2 | Otitis externa in mycoses |
| H66 | Suppurative and unspecified otitis media |
| H67.1 | Otitis media in viral diseases classified elsewhere |
| I02 | Rheumatic chorea |
| I30.1 | Infective pericarditis |
| I32.1 | Pericarditis in other infectious and parasitic diseases classified elsewhere |
| I33.0 | Acute and subacute infective endocarditis |
| I38 | Endocarditis, valve unspecified |
| I39 | Endocarditis and heart valve disorders in diseases classified elsewhere |
| I40.0 | Infective myocarditis |
| I40.9 | Acute myocarditis, unspecified |
| I41.0 | Myocarditis in bacterial diseases classified elsewhere |
| I41.1 | Myocarditis in viral diseases classified elsewhere |
| I68.1 | Cerebral arteritis in infectious and parasitic diseases classified elsewhere |
| I80.051 | Suppurative phlebitis of superficial vessels of lower extremities |
| I80.251 | Suppurative phlebitis of other deep vessels of lower extremities |
| I80.351 | Suppurative phlebitis of lower extremities, unspecified |
| I98.0 | Cardiovascular syphilis |
| I98.1 | Cardiovascular disorders in other infectious and parasitic diseases classified elsewhere |
| J00 | Acute nasopharyngitis [common cold] |
| J01 | Acute sinusitis |
| J01.0 | Acute maxillary sinusitis |
| J01.1 | Acute frontal sinusitis |
| J01.2 | Acute ethmoidal sinusitis |
| J01.3 | Acute sphenoidal sinusitis |
| J01.4 | Acute pansinusitis |
| J01.8 | Other acute sinusitis |
| J01.9 | Acute sinusitis, unspecified |
| J02 | Acute pharyngitis |
| J02.0 | Streptococcal pharyngitis |
| J02.8 | Acute pharyngitis due to other specified organisms |
| J02.9 | Acute pharyngitis, unspecified |
| J03 | Acute tonsillitis |
| J03.0 | Streptococcal tonsillitis |
| J03.8 | Acute tonsillitis due to other specified organisms |
| J03.9 | Acute tonsillitis, unspecified |
| J04 | Acute laryngitis and tracheitis |
| J04.0 | Acute laryngitis |
| J04.1 | Acute tracheitis |
| J04.2 | Acute laryngotracheitis |
| J05 | Acute obstructive laryngitis [croup] and epiglottitis |
| J05.0 | Acute obstructive laryngitis [croup] |
| J05.1 | Acute epiglottitis |
| J06 | Acute upper respiratory infections of multiple and unspecified sites |
| J06.0 | Acute laryngopharyngitis |
| J06.8 | Other acute upper respiratory infections of multiple sites |
| J06.9 | Acute upper respiratory infection, unspecified |
| J09 | Influenza due to identified avian influenza virus |
| J10 | Influenza due to identified influenza virus |
| J10.0 | Influenza with pneumonia, influenza virus identified |
| J10.1 | Influenza with other respiratory manifestations, influenza virus identified |
| J10.8 | Influenza with other manifestations, influenza virus identified |
| J11 | Influenza, virus not identified |
| J11.0 | Influenza with pneumonia, virus not identified |
| J11.1 | Influenza with other respiratory manifestations, virus not identified |
| J11.8 | Influenza with other manifestations, virus not identified |
| J12 | Viral pneumonia, not elsewhere classified |
| J12.0 | Adenoviral pneumonia |
| J12.1 | Respiratory syncytial virus pneumonia |
| J12.2 | Parainfluenza virus pneumonia |
| J12.8 | Other viral pneumonia |
| J12.9 | Viral pneumonia, unspecified |
| J13 | Pneumonia due to Streptococcus pneumoniae |
| J14 | Pneumonia due to Hemophilus influenzae |
| J15 | Bacterial pneumonia, not elsewhere classified |
| J15.0 | Pneumonia due to Klebsiella pneumoniae |
| J15.1 | Pneumonia due to Pseudomonas |
| J15.2 | Pneumonia due to staphylococcus |
| J15.3 | Pneumonia due to streptococcus, group B |
| J15.4 | Pneumonia due to other streptococci |
| J15.5 | Pneumonia due to Escherichia coli |
| J15.6 | Pneumonia due to other aerobic Gram<U+2011>negative bacteria |
| J15.7 | Pneumonia due to Mycoplasma pneumoniae |
| J15.8 | Other bacterial pneumonia |
| J15.9 | Bacterial pneumonia, unspecified |
| J16 | Pneumonia due to other infectious organisms, not elsewhere classified |
| J16.0 | Chlamydial pneumonia |
| J16.8 | Pneumonia due to other specified infectious organisms |
| J18 | Pneumonia, organism unspecified |
| J18.0 | Bronchopneumonia, unspecified |
| J18.1 | Lobar pneumonia, unspecified |
| J18.2 | Hypostatic pneumonia, unspecified |
| J18.8 | Other pneumonia, organism unspecified |
| J18.9 | Pneumonia, unspecified |
| J20 | Acute bronchitis |
| J20.0 | Acute bronchitis due to Mycoplasma pneumoniae |
| J20.1 | Acute bronchitis due to Hemophilus influenzae |
| J20.2 | Acute bronchitis due to streptococcus |
| J20.3 | Acute bronchitis due to coxsackie virus |
| J20.4 | Acute bronchitis due to parainfluenza virus |
| J20.5 | Acute bronchitis due to respiratory syncytial virus |
| J20.6 | Acute bronchitis due to rhinovirus |
| J20.7 | Acute bronchitis due to echovirus |
| J20.8 | Acute bronchitis due to other specified organisms |
| J20.9 | Acute bronchitis, unspecified |
| J21 | Acute bronchiolitis |
| J21.0 | Acute bronchiolitis due to respiratory syncytial virus |
| J21.8 | Acute bronchiolitis due to other specified organisms |
| J21.9 | Acute bronchiolitis, unspecified |
| J22 | Unspecified acute lower respiratory infection |
| J34.0 | Abscess, furuncle and carbuncle of nose |
| J36 | Peritonsillar abscess |
| J39.0 | Retropharyngeal and parapharyngeal abscess |
| J39.1 | Other abscess of pharynx |
| J40 | Bronchitis, not specified as acute or chronic |
| J41 | Simple and mucopurulent chronic bronchitis |
| J41.0 | Simple chronic bronchitis |
| J41.1 | Mucopurulent chronic bronchitis |
| J41.8 | Mixed simple and mucopurulent chronic bronchitis |
| J42 | Unspecified chronic bronchitis |
| J44.0 | Chronic obstructive pulmonary disease with acute lower respiratory infection |
| J44.1 | Chronic obstructive pulmonary disease with acute exacerbation, unspecified |
| J85 | Abscess of lung and mediastinum |
| J85.0 | Gangrene and necrosis of lung |
| J85.1 | Abscess of lung with pneumonia |
| J85.2 | Abscess of lung without pneumonia |
| J85.3 | Abscess of mediastinum |
| J86 | Pyothorax |
| J86.0 | Pyothorax with fistula |
| J86.9 | Pyothorax without fistula |
| K04.0 | Pulpitis |
| K04.4 | Acute apical periodontitis of pulpal origin |
| K10.2 | Inflammatory conditions of jaws |
| K12 | Stomatitis and related lesions |
| K14.0 | Glossitis |
| K22.3 | Perforation of oesophagus |
| K25.1 | Acute gastric ulcer with perforation |
| K25.2 | Acute gastric ulcer with both haemorrhage and perforation |
| K26.1 | Acute duodenal ulcer with perforation |
| K26.2 | Acute duodenal ulcer with both haemorrhage and perforation |
| K27.1 | Acute peptic ulcer with perforation, site unspecified |
| K27.2 | Acute peptic ulcer with both haemorrhage and perforation, site unspecified |
| K28.1 | Acute gastrojejunal ulcer with perforation |
| K28.2 | Acute gastrojejunal ulcer with both haemorrhage and perforation |
| K31.6 | Fistula of stomach and duodenum |
| K35 | Acute appendicitis |
| K35.0 | Acute appendicitis with generalized peritonitis |
| K35.1 | Acute appendicitis with peritoneal abscess |
| K35.9 | Acute appendicitis, unspecified |
| K36 | Other appendicitis |
| K37 | Unspecified appendicitis |
| K40.1 | Bilateral inguinal hernia, with gangrene |
| K40.4 | Unilateral or unspecified inguinal hernia, with gangrene |
| K41.4 | Unilateral or unspecified femoral hernia, with gangrene |
| K42.1 | Umbilical hernia, with gangrene |
| K43.1 | Ventral hernia, with gangrene |
| K44.1 | Diaphragmatic hernia, with gangrene |
| K45.1 | Other specified abdominal hernia, with gangrene |
| K46.1 | Unspecified abdominal hernia, with gangrene |
| K57.0 | Diverticular disease of small intestine with perforation and abscess |
| K57.2 | Diverticular disease of large intestine with perforation and abscess |
| K57.4 | Diverticular disease of both small and large intestine with perforation and abscess |
| K57.8 | Diverticular disease of intestine, part unspecified, with perforation and abscess |
| K60.3 | Anal fistula |
| K60.4 | Rectal fistula |
| K60.5 | Anorectal fistula |
| K61 | Abscess of anal and rectal regions |
| K63.0 | Abscess of intestine |
| K63.1 | Perforation of intestine (nontraumatic) |
| K63.2 | Fistula of intestine |
| K65 | Peritonitis |
| K65.0 | Acute peritonitis |
| K65.8 | Other peritonitis |
| K65.9 | Peritonitis, unspecified |
| K75.0 | Abscess of liver |
| K75.1 | Phlebitis of portal vein |
| K77.0 | Liver disorders in infectious and parasitic diseases classified elsewhere |
| K80.0 | Calculus of gallbladder with acute cholecystitis |
| K80.1 | Calculus of gallbladder with other cholecystitis |
| K80.3 | Calculus of bile duct with cholangitis |
| K80.4 | Calculus of bile duct with cholecystitis |
| K81 | Cholecystitis |
| K82.2 | Perforation of gallbladder |
| K82.3 | Fistula of gallbladder |
| K83.0 | Cholangitis |
| K83.2 | Perforation of bile duct |
| K83.3 | Fistula of bile duct |
| K85 | Acute pancreatitis |
| K85.0 | Idiopathic acute pancreatitis |
| K85.1 | Biliary acute pancreatitis |
| K85.2 | Alcohol-induced acute pancreatitis |
| K85.3 | Drug-induced acute pancreatitis |
| K85.8 | Other acute pancreatitis |
| K85.9 | Acute pancreatitis, unspecified |
| M49.2 | Enterobacterial spondylitis |
| M60.0 | Infective myositis |
| M65.1 | Other infective (teno)synovitis |
| M71.1 | Other infective bursitis |
| M72.6 | Necrotising fasciitis |
| M86 | Osteomyelitis |
| M90.1 | Periositis in other infectious diseases classified elsewhere |
| N13.6 | Pyonephrosis |
| N15.1 | Renal and perinephric abscess |
| N30.0 | Acute cystitis |
| N30.3 | Trigonitis |
| N30.8 | Other cystitis |
| N32.1 | Vesicointestinal fistula |
| N32.3 | Diverticulum of bladder |
| N34.0 | Urethral abscess |
| N34.2 | Other urethritis |
| N37.0 | Urethritis in diseases classified elsewhere |
| N39.0 | Urinary tract infection, site not specified |
| N41.0 | Acute prostatitis |
| N41.2 | Abscess of prostate |
| N41.3 | Prostatocystitis |
| N43.1 | Infected hydrocele |
| N45 | Orchitis and epididymitis |
| N48.2 | Other inflammatory disorders of penis |
| O41.1 | Infection of amniotic sac and membranes |
| O74.0 | Aspiration pneumonitis due to anaesthesia during labour and delivery |
| P24 | Neonatal aspiration syndromes |
| P77 | Necrotizing entrocolitis of fetus and newborn |
| P78.0 | Perinatal intestinal perforation |
| P78.1 | Other neonatal peritonitis |
| R02 | Gangrene, not elsewhere classified |
| R10.0 | Acute abdomen |
| R65.0 | Systemic inflammatory response syndrome of infectious origin without organ failure |
| R65.1 | Systemic inflammatory response syndrome of infectious origin with organ failure |
| T80.2 | Infections following infusion, transfusion and therapeutic injection |
| T81.4 | Infection following a procedure, not elsewhere classified |
| T82.6 | Infection and inflammatory reaction due to cardiac valve prosthesis |
| T82.7 | Infection and inflammatory reaction due to other cardiac and vascular devices, implants and grafts |
| T83.5 | Infection and inflammatory reaction due to prosthetic device, implant and graft in urinary tract |
| T84.5 | Infection and inflammatory reaction due to internal joint prosthesis |
| T84.6 | Infection and inflammatory reaction due to internal fixation device [any site] |
| T85.7 | Infection and inflammatory reaction due to other internal prosthetic devices, implants and grafts |
| T87.4 | Infection of amputation stump |
| T88.0 | Infection following immunization |

ICD-10 codes of infection were derived from the modified Angus criteria^1^, with the addition of codes of chronic bronchitis (J40, J41, J41.0, J41.1, J41.8, J42, J44.0, J44.1), which probably indicated an infectious cause of acute exacerbation of chronic bronchitis and were validated in a previous study.^2^

# Table S3. International Classification of Diseases (ICD) codes for underlying causes of sepsis-related deaths.

| **Underlying cause of death** | **ICD 10 codes** |
| --- | --- |
| Lower respiratory infections | J12-J18.9, J20-J22.9 |
| Neoplasms | C00-C97.9 |
| Cardiovascular diseases | I00-I25.9 |
| Cerebrovascular disease | I60-I69.9 |
| Chronic respiratory diseases | J30-J98.9 |
| Digestive diseases | K20-K92.9 |
| Diabetes mellitus | E10-E14.9 |
| Chronic kidney disease | N00-N19.9 |

Underlying causes of sepsis-related deaths are reported according to the underlying GBD causes, 2020 Year book of health in the People’s Republic of China, and National Standard Classification and Codes of Diseases.^3–5^

# Table S4. Number of deaths, age-standardized mortality and YLLs related to sepsis in China, for all ages, both sexes, per year from 2006 to 2020.

|  | **2006** | **2007** | **2008** | **2009** | **2010** | **2011** | **2012** | **2013** | **2014** | **2015** | **2016** | **2017** | **2018** | **2019** | **2020** |
| --- | --- | --- | --- | --- | --- | --- | --- | --- | --- | --- | --- | --- | --- | --- | --- |
| **Number of deaths (95%CI), DSPs** | | | | | | | | | | | | | | | |
| Both sexes | 49536 (49100-49972) | 54064 (53608-54520) | 52034 (51587-52481) | 58539 (58065-59013) | 60877 (60394-61360) | 52888 (52437-53339) | 57334 (56865-57803) | 196878 (196009-197747) | 233243 (232297-234189) | 248250 (247274-249226) | 263577 (262571-264583) | 262935 (261930-263940) | 271095 (270075-272115) | 272698 (271675-273721) | 237326 (236371-238281) |
| Male | 27562 (27237-27887) | 30539 (30197-30881) | 30198 (29858-30538) | 33192 (32835-33549) | 35209 (34841-35577) | 30130 (29790-30470) | 32468 (32115-32821) | 112456 (111799-113113) | 134468 (133750-135186) | 143087 (142346-143828) | 152319 (151554-153084) | 153322 (152555-154089) | 158827 (158046-159608) | 160404 (159619-161189) | 141094 (140358-141830) |
| Female | 21974 (21684-22264) | 23525 (23224-23826) | 21836 (21546-22126) | 25347 (25035-25659) | 25668 (25354-25982) | 22758 (22462-23054) | 24866 (24557-25175) | 84422 (83853-84991) | 98775 (98159-99391) | 105163 (104528-105798) | 111258 (110604-111912) | 109613 (108964-110262) | 112268 (111611-112925) | 112294 (111637-112951) | 96232 (95624-96840) |
| **Age-standardized mortality per 100,000 population (95%CI)** | | | | | | | | | | | | | | | |
| Both sexes | 130.2 (129.4-131) | 143.1 (142.2-143.9) | 133.7 (132.9-134.5) | 150.9 (150-151.7) | 156.2 (155.4-157.1) | 118.9 (118.1-119.6) | 100.1 (99.4-100.8) | 86 (85.6-86.3) | 95.9 (95.6-96.3) | 101.5 (101.2-101.8) | 97.4 (97.1-97.8) | 96.3 (95.9-96.6) | 93.3 (92.9-93.6) | 96.7 (96.4-97) | 76.6 (76.3-76.9) |
| Male | 161.3 (160.1-162.6) | 178.1 (176.8-179.4) | 169.7 (168.4-171) | 188.4 (187.1-189.8) | 200.6 (199.2-201.9) | 153.6 (152.4-154.8) | 126.2 (125.1-127.3) | 107.4 (106.9-107.9) | 120.6 (120-121.1) | 128.4 (127.9-129) | 124 (123.4-124.5) | 124.1 (123.5-124.6) | 121 (120.5-121.5) | 124 (123.5-124.5) | 101.6 (101.1-102) |
| Female | 106.6 (105.5-107.6) | 116.2 (115.1-117.3) | 105.9 (104.9-107) | 122.2 (121.1-123.3) | 122.9 (121.7-124) | 93.6 (92.6-94.5) | 78.9 (78-79.8) | 67.8 (67.4-68.2) | 74.7 (74.3-75.2) | 78.6 (78.2-79.1) | 74.9 (74.5-75.3) | 72.9 (72.5-73.3) | 69.9 (69.5-70.3) | 73.4 (73-73.9) | 56 (55.6-56.3) |
| **YLLs (95%CI), DSPs** | | | | | | | | | | | | | | | |
| Both sexes | 1094073 (1086303-1101843) | 1173687 (1165757-1181616) | 1214625 (1206375-1222875) | 1274390 (1266023-1282758) | 1322712 (1314260-1331164) | 1069381 (1061862-1076899) | 1093190 (1085587-1100794) | 3745637 (3731370-3759904) | 4394128 (4378819-4409437) | 4574409 (4558920-4589897) | 4781596 (4765792-4797400) | 4719807 (4704149-4735466) | 4817525 (4801712-4833339) | 4788592 (4772865-4804319) | 4113654 (4099171-4128136) |
| Male | 642948 (637027-648869) | 695345 (689281-701409) | 747785 (741362-754207) | 765524 (759077-771971) | 806227 (799694-812760) | 650599 (644732-656466) | 658727 (652816-664638) | 2297179 (2285985-2308373) | 2720955 (2708890-2733020) | 2827414 (2815237-2839590) | 2972195 (2959721-2984670) | 2957037 (2944644-2969430) | 3031242 (3018719-3043766) | 3035194 (3022659-3047729) | 2624359 (2612818-2635900) |
| Female | 451125 (446146-456104) | 478342 (473291-483393) | 466840 (461732-471948) | 508866 (503608-514125) | 516485 (511211-521759) | 418782 (414156-423408) | 434463 (429742-439185) | 1448458(1439727-1457189) | 1673173 (1663878-1682468) | 1746995 (1737559-1756432) | 1809400(1799837-1818964) | 1762770 (1753351-1772190) | 1786283 (1776783-1795783) | 1753398 (1744062-1762734) | 1489295 (1480691-1497899) |
| **Age-standardized YLLs per 100,000 population (95%CI)** | | | | | | | | | | | | | | | |
| Both sexes | 2172.7 (2169.4-2176) | 2321.2 (2317.9-2324.6) | 2237 (2233.7-2240.3) | 2386.5 (2383.1-2389.9) | 2440.6 (2437.2-2444) | 1846.1 (1843.2-1849.1) | 1704.4 (1701.6-1707.2) | 1471.2 (1469.9-1472.5) | 1652.5 (1651.1-1653.9) | 1719.6 (1718.2-1721) | 1662 (1660.7-1663.4) | 1631.8 (1630.4-1633.1) | 1590.7 (1589.3-1592) | 1563 (1561.7-1564.3) | 1271 (1269.8-1272.2) |
| Male | 2707.9 (2702.8-2713) | 2890 (2884.7-2895.2) | 2855 (2849.8-2860.2) | 2999.4 (2994.1-3004.7) | 3131 (3125.6-3136.4) | 2401.7 (2397.1-2406.4) | 2163.5 (2159.1-2167.9) | 1868.7 (1866.7-1870.8) | 2116.6 (2114.4-2118.8) | 2214.5 (2212.3-2216.8) | 2155.9 (2153.7-2158.1) | 2141 (2138.9-2143.2) | 2101 (2098.8-2103.1) | 2069 (2066.9-2071.1) | 1716.6 (1714.7-1718.6) |
| Female | 1715.3 (1711.2-1719.5) | 1828.6 (1824.3-1832.9) | 1692.4 (1688.3-1696.5) | 1854 (1849.7-1858.3) | 1849.1 (1844.8-1853.4) | 1382.1(1378.5-1385.7) | 1293.9(1290.4-1297.4) | 1100.8(1099.2-1102.5) | 1216.4(1214.7-1218.1) | 1260.6(1258.8-1262.3) | 1201.9(1200.3-1203.6) | 1159.6(1157.9-1161.2) | 1117.3(1115.7-1118.9) | 1097.9(1096.3-1099.5) | 868.8(867.4-870.2) |

95%CI, 95% confidence interval. DSPs, disease surveillance points. YLLs, years of life lost.

# Table S5. Number of deaths and mortality related to sepsis in China, 2006-2020, by age.

|  | **Sepsis-related deaths (95%CI)** | | | **Mortality per 100,000 population (95%CI)** | | |
| --- | --- | --- | --- | --- | --- | --- |
|  | **DSPs, 2020** | **Percentage change (%), 2006-2020** | **National estimate, 2020** | **2020** | **Percentage change (%), 2006-2020** | **AAPC (%, 95%CI), 2006-2020** |
| **Both sexes** |  |  |  |  |  |  |
| <1 | 944 (884-1004) | -23.4 | 3176 (2974-3379) | 26.5(24.8-28.2) | -79.7 | -9.2 (-13.5 to -4.7) |
| 1-4 | 399 (360-438) | 1.3 | 1710 (1542-1878) | 2.6(2.3-2.9) | -75.5 | -7.7 (-15.1 to 0.3) |
| 5-14 | 495 (451-539) | 112.4 | 2423 (2210-2637) | 1.4(1.3-1.5) | -44.0 | -2.2 (-9.9 to 6.2) |
| 15-24 | 611 (563-659) | 107.1 | 2578 (2374-2783) | 1.7(1.6-1.9) | -20.7 | -0.8 (-9.2 to 8.4) |
| 25-34 | 1356 (1284-1428) | 220.6 | 5363 (5078-5649) | 2.5(2.4-2.6) | -31.4 | -1.5 (-7.9 to 5.3) |
| 35-44 | 2529 (2430-2628) | 144.8 | 9925 (9539-10312) | 5.2(5-5.4) | -30.9 | -2.3 (-4 to -0.5) |
| 45-54 | 8031 (7855-8207) | 302.0 | 31658 (30965-32350) | 13.4(13.2-13.7) | -30.2 | -3 (-7.2 to 1.4) |
| 55-64 | 17891 (17629-18153) | 306.2 | 73110 (72039-74181) | 41.8(41.2-42.4) | -36.7 | -3.4 (-5.8 to -0.9) |
| 65-74 | 45090 (44674-45506) | 282.9 | 195732 (193927-197537) | 158.4(156.9-159.8) | -44.3 | -3.3 (-4.4 to -2.2) |
| 75-84 | 82067 (81507-82627) | 346.5 | 362457 (359986-364928) | 702.1(697.4-706.9) | -31.3 | -2.8 (-3.8 to -1.8) |
| ≥85 | 77913 (77373-78453) | 731.7 | 391121 (388409-393832) | 2536.8(2519.2-2554.4) | -46.2 | -3 (-9.3 to 3.8) |
| **Male** |  |  |  |  |  |  |
| <1 | 545 (499-591) | -23.0 | 1846 (1691-2001) | 29.2 (26.8-31.7) | -78.6 | -9.2 (-13.87 to -4.36) |
| 1-4 | 220 (191-249) | 0.5 | 935 (812-1059) | 2.7 (2.3-3.1) | -76.5 | -8.1 (-16.01 to 0.67) |
| 5-14 | 284 (251-317) | 127.2 | 1375 (1215-1535) | 1.5 (1.3-1.6) | -41.7 | -1.9 (-10.26 to 7.36) |
| 15-24 | 371 (333-409) | 102.7 | 1556 (1398-1715) | 2.0 (1.8-2.2) | -26.4 | -0.9 (-9.67 to 8.78) |
| 25-34 | 938 (878-998) | 243.6 | 3779 (3537-4021) | 3.4 (3.2-3.6) | -28.0 | -1.1 (-7.28 to 5.51) |
| 35-44 | 1819 (1735-1903) | 183.8 | 7228 (6896-7561) | 7.3 (7.0-7.7) | -21.0 | -1.6 (-3.33 to 0.07) |
| 45-54 | 5786 (5637-5935) | 337.7 | 22785 (22198-23372) | 19.1 (18.6-19.6) | -24.7 | -2.4 (-7.08 to 2.56) |
| 55-64 | 13062 (12838-13286) | 368.8 | 53312 (52398-54226) | 60.8 (59.8-61.8) | -26.7 | -2.4 (-4.69 to -0.01) |
| 65-74 | 30707 (30364-31050) | 319.3 | 133860 (132364-135355) | 221.3 (218.8-223.7) | -39.0 | -2.8 (-3.84 to -1.65) |
| 75-84 | 49067 (48635-49499) | 388.9 | 220490 (218548-222432) | 922.2 (914.1-930.3) | -27.7 | -2.7 (-3.70 to -1.68) |
| ≥85 | 38295 (37918-38672) | 871.0 | 194446 (192529-196362) | 3187.5 (3156.1-3218.9) | -43.2 | -2.7 (-8.46 to 3.5) |
| **Female** |  |  |  |  |  |  |
| <1 | 399 (360-438) | -23.9 | 1333 (1202-1463) | 23.5 (21.2-25.8) | -79.2 | -9.8 (-13.24 to -6.27) |
| 1-4 | 179 (153-205) | 2.3 | 774 (661-888) | 2.5 (2.1-2.8) | -74.2 | -7.4 (-14.43 to 0.26) |
| 5-14 | 211 (183-239) | 95.4 | 1046 (905-1188) | 1.3 (1.1-1.5) | -46.7 | -4.3 (-6.73 to -1.78) |
| 15-24 | 240 (210-270) | 114.3 | 1020 (891-1149) | 1.5 (1.3-1.7) | -12.9 | -0.9 (-9.62 to 8.70) |
| 25-34 | 418 (378-458) | 178.7 | 1621 (1466-1777) | 1.6 (1.4-1.7) | -39.1 | -4.6 (-7.07 to -2.11) |
| 35-44 | 710 (658-762) | 81.1 | 2751 (2548-2953) | 2.9 (2.7-3.2) | -48.3 | -3.8 (-5.77 to -1.70) |
| 45-54 | 2245 (2152-2338) | 232.1 | 8859 (8492-9225) | 7.6 (7.3-8.0) | -41.8 | -4.3 (-8.05 to -0.33) |
| 55-64 | 4829 (4693-4965) | 198.5 | 19757 (19200-20314) | 22.7 (22.0-23.3) | -53.6 | -5.4 (-8.23 to -2.55) |
| 65-74 | 14383 (14148-14618) | 223.1 | 62185 (61169-63200) | 98.6 (97.0-100.2) | -53.0 | -4.4 (-5.58 to -3.16) |
| 75-84 | 33000 (32645-33355) | 295.5 | 143623 (142077-145168) | 518.3 (512.7-523.8) | -37.1 | -3.2 (-4.10 to -2.21) |
| ≥85 | 39618 (39232-40004) | 630.4 | 197415 (195492-199338) | 2118.7 (2098.1-2139.4) | -49.9 | -3.4 (-10.25 to 4.01) |

95%CI, 95% confidence interval. AAPC, average annual percentage change. DSPs, disease surveillance points.

# Table S6. Number of deaths, age-standardized mortality and YLLs related to sepsis, 2006-2020, by province of China.

|  | **Sepsis-related deaths (95%CI)** | | | **Age-standardized mortality per 100,000 population (95%CI)** | | | **Age-standardized YLLs per 100,000 population (95%CI)** | | |
| --- | --- | --- | --- | --- | --- | --- | --- | --- | --- |
| **Province** | **DSPs, 2020** | **Percentage change (%), 2006-2020** | **Provincial estimate, 2020** | **2020** | **Percentage change (%), 2006-2020** | **AAPC (%, 95%CI),**  **2006-2020** | **2020** | **Percentage change (%), 2006-2020** | **AAPC (%, 95%CI),**  **2006-2020** |
| Anhui | 8574  (8393-8755) | 341.3 | 33333  (32628-34039) | 54.7  (53.5-55.8) | -52.6 | -5.4  (-7.1 to -3.6) | 860  (855.4-864.5) | -50.5 | -4.6  (-6.1 to -3.2) |
| Beijing | 4764  (4629-4899) | 1537.1 | 13229  (12853-13604) | 70.1  (68.2-71.9) | 118.8 | 6.8  (-0.4 to 14.6) | 1075.3  (1068.1-1082.5) | 144.4 | 7.9  (1.3 to 14.8) |
| Chongqing | 11478  (11268-11688) | 391.4 | 36827  (36154-37500) | 109.7  (107.7-111.8) | -40.5 | -2.5  (-8 to 3.4) | 1860.6  (1852.2-1869) | -41.3 | -3.7  (-5.2 to -2.2) |
| Fujian | 5862  (5712-6012) | 297.7 | 22895  (22309-23481) | 75.1  (73.4-76.7) | -42.4 | -2.4  (-7.4 to 2.8) | 1160.2  (1153.8-1166.6) | -46.8 | -3.1  (-7.3 to 1.3) |
| Gansu | 6892  (6729-7055) | 501.9 | 21458  (20952-21965) | 111  (108.7-113.3) | -44.4 | -5.4  (-8.4 to -2.2) | 1850.1  (1840.7-1859.4) | -45.0 | -5.3  (-8 to -2.6) |
| Guangdong | 20458  (20178-20738) | 681.7 | 91675  (90419-92931) | 93.9  (92.8-95) | -42.6 | -3.5  (-7.8 to 1) | 1587.7  (1583.1-1592.3) | -40.8 | -2.7  (-5.5 to 0.2) |
| Guangxi | 10585  (10383-10787) | 668.7 | 54633  (53593-55673) | 142.2  (139.9-144.6) | 62.6 | 2.5  (-1.4 to 6.6) | 2329.3  (2319.8-2338.8) | 41.6 | 2.1  (-1.2 to 5.5) |
| Guizhou | 9173  (8985-9361) | 498.4 | 38406  (37621-39192) | 132  (129.6-134.3) | -43.0 | -4.1  (-6.9 to -1.3) | 2463.9  (2453.8-2473.9) | -44.0 | -3.7  (-5.7 to -1.6) |
| Hainan | 1991  (1904-2078) | 384.4 | 6989  (6682-7296) | 85.9  (82.5-89.3) | -28.8 | -2.3  (-6.7 to 2.2) | 1360.6  (1347.2-1374) | -32.5 | -2.3  (-5.7 to 1.2) |
| Hebei | 6271  (6116-6426) | 407.8 | 29811  (29073-30548) | 45.4  (44.3-46.4) | -41.6 | -1.6  (-4.3 to 1.1) | 789.7  (785.3-794.1) | -40.8 | -0.9  (-3.6 to 2) |
| Heilongjiang | 6263  (6108-6418) | 509.8 | 20016  (19520-20511) | 62.6  (61-64.1) | -35.7 | -2.3  (-10 to 5.9) | 1201.8  (1195-1208.5) | -19.4 | -0.3  (-7 to 6.9) |
| Henan | 9258  (9069-9447) | 221.4 | 43307  (42425-44189) | 55.3  (54.3-56.3) | -45.3 | -3.8  (-5.7 to -1.9) | 915.5  (911.5-919.6) | -49.7 | -3.9  (-5.5 to -2.2) |
| Hubei | 8296  (8118-8474) | 543.1 | 38526  (37698-39355) | 72.3  (70.8-73.8) | -5.7 | 0.4  (-2.3 to 3.1) | 1227.2  (1221.1-1233.3) | -1.5 | 1.2  (-1.7 to 4.2) |
| Hunan | 14883  (14644-15122) | 186.6 | 54115  (53246-54984) | 84.7  (83.4-86) | -63.3 | -7  (-9.1 to -4.8) | 1474.4  (1468.8-1479.9) | -59.7 | -5.7  (-7.7 to -3.8) |
| Inner Mongolia | 3668  (3549-3787) | 578.0 | 15648  (15142-16155) | 89.5  (87.1-92) | -14.2 | -0.8  (-8.1 to 7.1) | 1495.8  (1485.8-1505.9) | -9.6 | -0.1  (-7.3 to 7.5) |
| Jiangsu | 15828  (15582-16074) | 796.3 | 59515  (58588-60442) | 68.7  (67.6-69.8) | -35.9 | -0.3  (-2.5 to 2) | 947.3  (943.3-951.3) | -34.5 | -1.8  (-8.2 to 5.1) |
| Jiangxi | 6702  (6542-6862) | 571.5 | 36157  (35292-37022) | 99.8  (97.6-101.9) | -2.1 | -1.3  (-3.5 to 0.9) | 1555.3  (1546.9-1563.6) | -4.7 | 0.2  (-6.9 to 7.7) |
| Jilin | 2556  (2457-2655) | 276.4 | 9299  (8939-9660) | 39  (37.5-40.5) | -38.0 | -3.6  (-10.4 to 3.7) | 775.1  (768.4-781.8) | -25.6 | -2.2  (-7.3 to 3.2) |
| Liaoning | 4945  (4807-5083) | 200.8 | 19762  (19212-20313) | 40.6  (39.4-41.8) | -61.6 | -5.6  (-7.4 to -3.7) | 715.9  (710.8-720.9) | -52.6 | -4.1  (-5.8 to -2.5) |
| Ningxia | 1418  (1344-1492) | 293.9 | 2752  (2608-2895) | 63.4  (60.9-66) | -69.5 | -7  (-10 to -4) | 1035.1  (1024.8-1045.4) | -65.3 | -6.1  (-8.6 to -3.6) |
| Qinghai | 1407  (1334-1480) | 218.3 | 4711  (4465-4957) | 123.5  (118.3-128.6) | -51.0 | -4  (-9 to 1.3) | 2256.6  (2234.7-2278.5) | -49.0 | -3.8  (-7.3 to -0.2) |
| Shaanxi | 2786  (2683-2889) | 272.0 | 21053  (20271-21835) | 63.8  (61.6-66) | -52.8 | -4.6  (-7.1 to -2.1) | 1091.8  (1082.9-1100.7) | -54.3 | -4.5  (-6.8 to -2.1) |
| Shandong | 13086  (12862-13310) | 549.8 | 52021  (51130-52912) | 45.3  (44.5-46.1) | -27.7 | -2.2  (-4.6 to 0.2) | 749.4  (746.1-752.8) | -21.0 | -0.8  (-3.1 to 1.6) |
| Shanghai | 6732  (6571-6893) | 1119.6 | 18794  (18346-19243) | 61.5  (59.9-63.1) | -3.3 | -1.6  (-4.2 to 1) | 897.7  (891.5-903.9) | 7.5 | -0.7  (-3.5 to 2.2) |
| Shanxi | 3059  (2951-3167) | 131.6 | 14185  (13682-14687) | 50.2  (48.6-51.8) | -61.7 | -5.2  (-11 to 1) | 820.7  (814.3-827.2) | -61.7 | -5.4  (-10.3 to -0.3) |
| Sichuan | 23087  (22789-23385) | 437.4 | 112302  (110854-113750) | 124.2  (122.6-125.9) | -31.3 | -2.2  (-3.9 to -0.4) | 2043.5  (2036.8-2050.2) | -40.7 | -2.9  (-4.4 to -1.4) |
| Tianjin | 2892  (2787-2997) | 261.5 | 6051  (5831-6272) | 43.6  (42-45.2) | -57.9 | -5  (-9.1 to -0.7) | 722.8  (716.3-729.2) | -48.8 | -3.8  (-7.2 to -0.2) |
| Tibet | 466  (424-508) | 163.3 | 1880  (1710-2051) | 106  (99.2-112.7) | 10.5 | 0.4  (-5.4 to 6.6) | 2288.3  (2257.5-2319.1) | 1.5 | -0.6  (-5.8 to 4.9) |
| Xinjiang | 3646  (3528-3764) | 107.8 | 18371  (17775-18967) | 122.8  (119.8-125.9) | -36.9 | -3.7  (-6.1 to -1.2) | 2262.8  (2250-2275.7) | -48.5 | -5  (-7.1 to -2.8) |
| Yunnan | 10569  (10368-10770) | 231.7 | 51109  (50135-52083) | 151.2  (148.8-153.7) | -48.4 | -4.4  (-7.6 to -1.2) | 2591.3  (2581.3-2601.2) | -53.3 | -5.2  (-7.6 to -2.8) |
| Zhejiang | 9731  (9538-9924) | 177.8 | 47981  (47028-48934) | 72.7  (71.2-74.1) | -68.3 | -8.5  (-10.6 to -6.3) | 1027  (1021.5-1032.5) | -66.1 | -7.9  (-9.7 to -6.2) |

95%CI, 95% confidence interval. AAPC, average annual percentage change. DSPs, disease surveillance points. YLLs, years of life lost.

# Fig. S1. Percentage of all sepsis-related deaths for (A) male and (B) female in China, 2006-2020, by age.


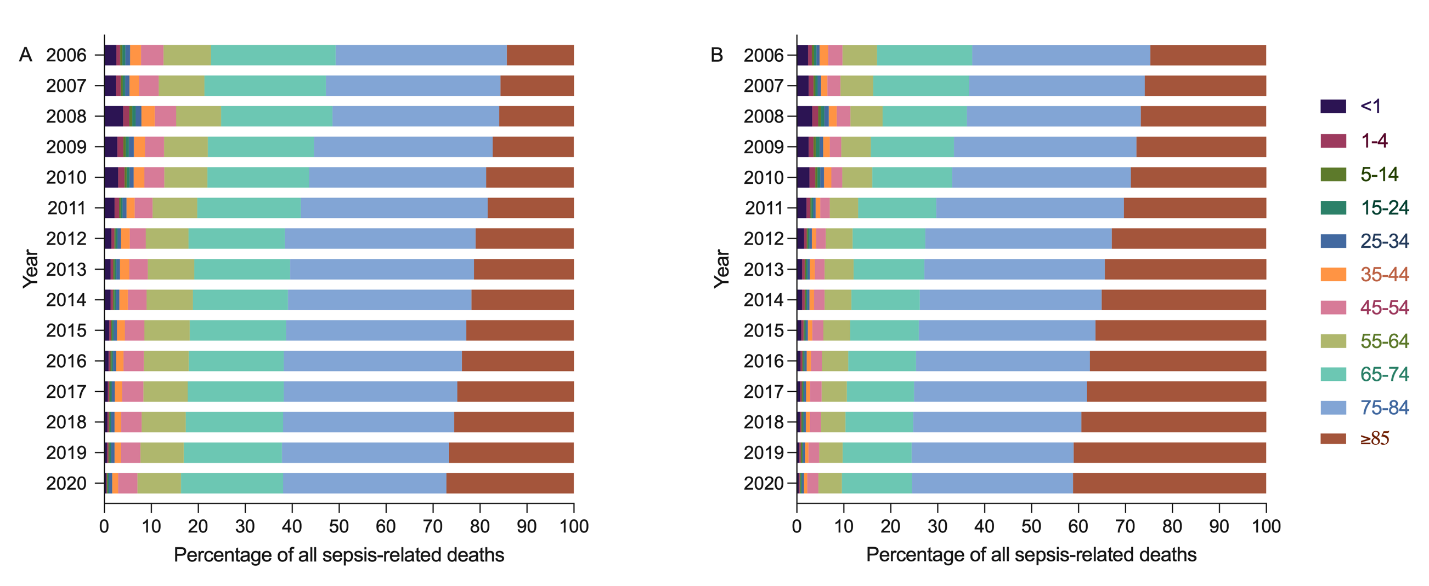


# Fig. S2. Age-standardized sepsis-related mortality per 100,000 population in China, by underlying cause of death.

Shading indicates the 95% confidence interval.


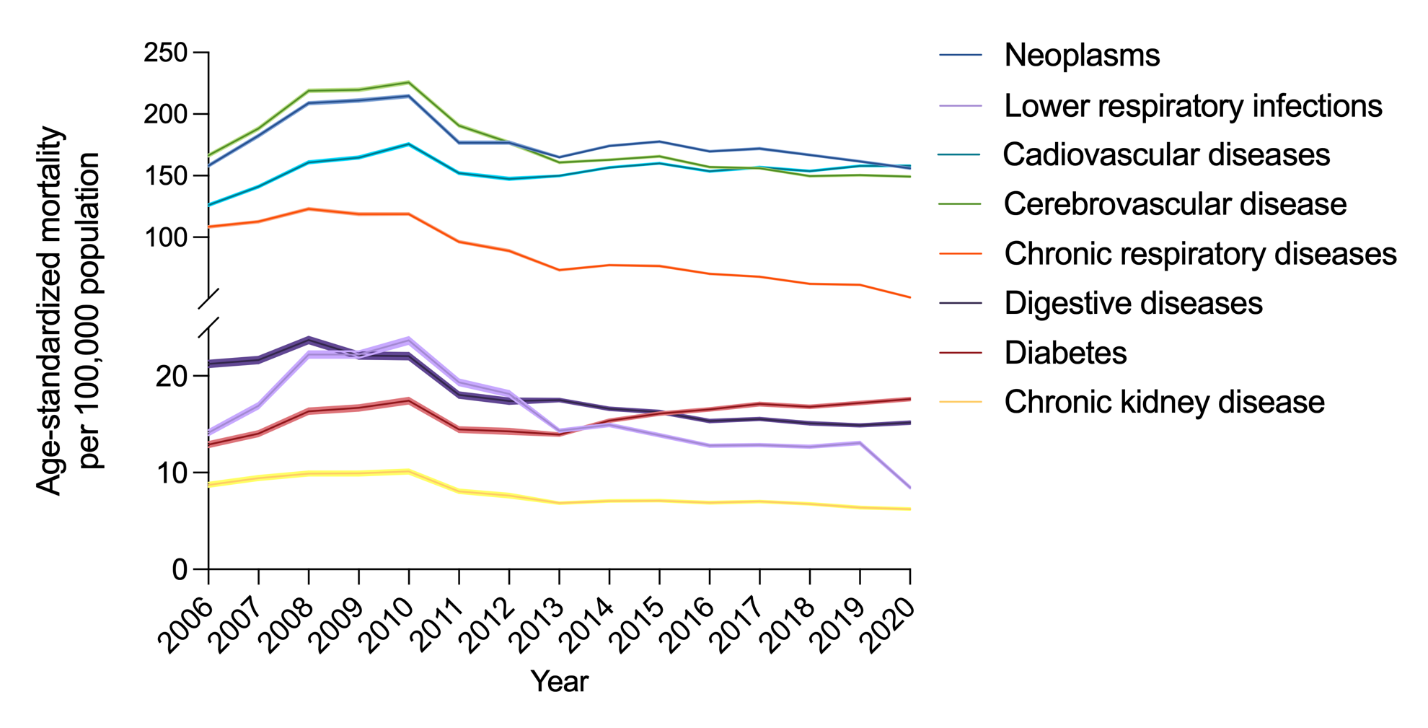


# Fig. S3. Place of death of decedents with sepsis in China, 2006-2020.


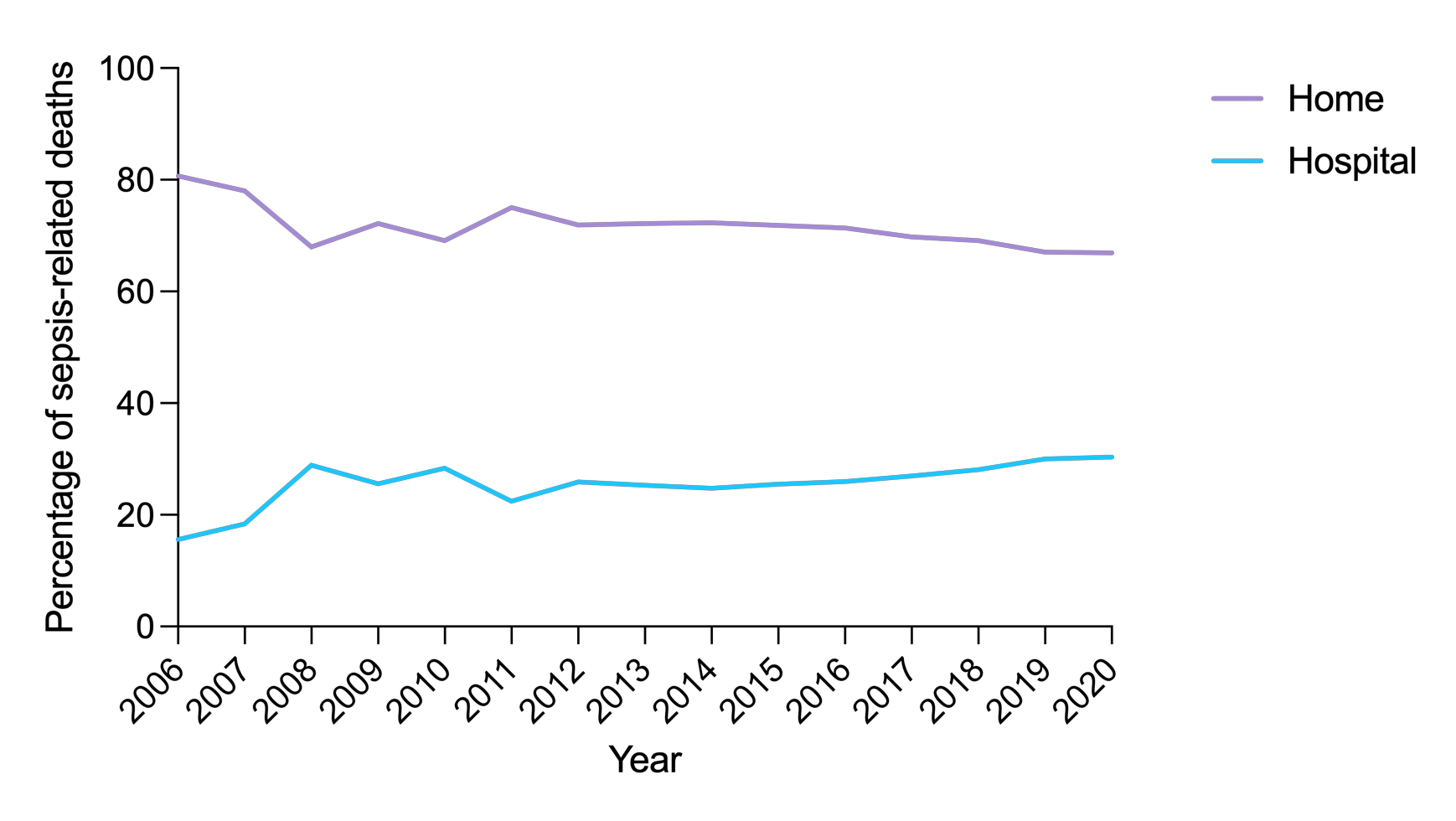


**Fig. S4. Place of sepsis-related death in China, by province.**

(A) Place of sepsis-related death in 2020 by province (Spearman ρ = -0.57, p < 0.001). (B) Change of percentage of sepsis-related death at home from 2006 to 2020 by province (Spearman ρ = -0.46, p = 0.02). Provinces are ranked by SDI from the lowest at the top to the highest at the bottom.


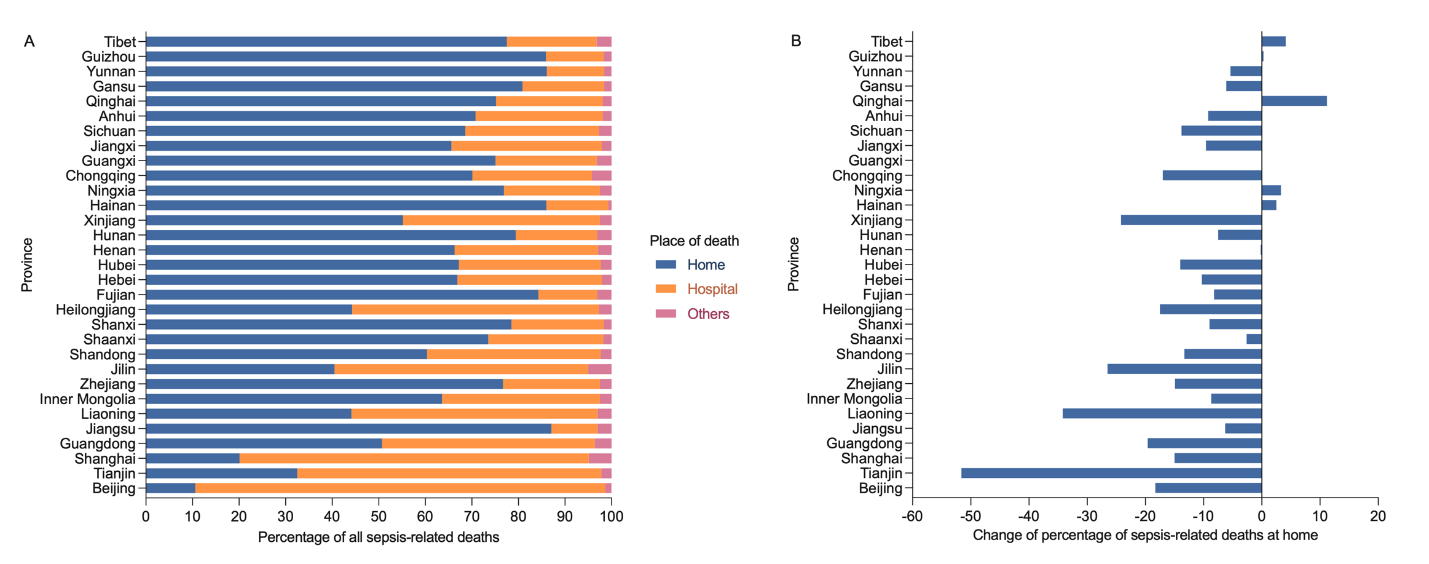


# Fig. S5. Age-standardized sepsis-related mortality and YLLs per 100,000 population in China, 2006-2020, by province.

(A) Age-standardized mortality in 2006. (B) Age-standardized mortality in 2020. (C) Age-standardized YLLs in 2006. (D) Age-standardized YLLs in 2020. YLLs, years of life lost. Results were reported as rates per 100,000 population.


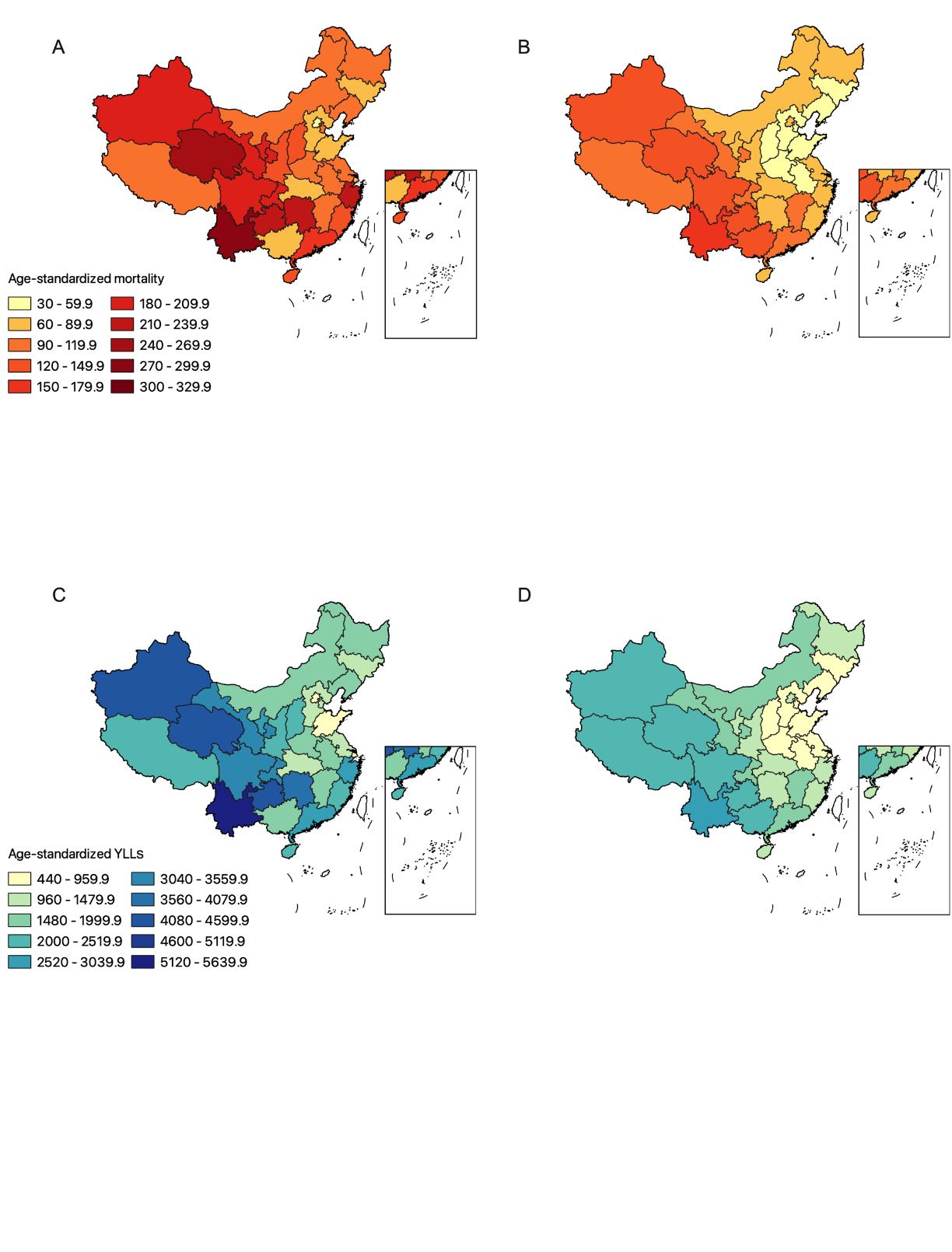


# Fig. S6. Age-standardized sepsis-related mortality and YLLs for each year 2006-2020, by province of China.

(A) Age-standardized mortality. (B) Age-standardized YLLs. YLLs, years of life lost. Results were reported as rates per 100,000 population.

**
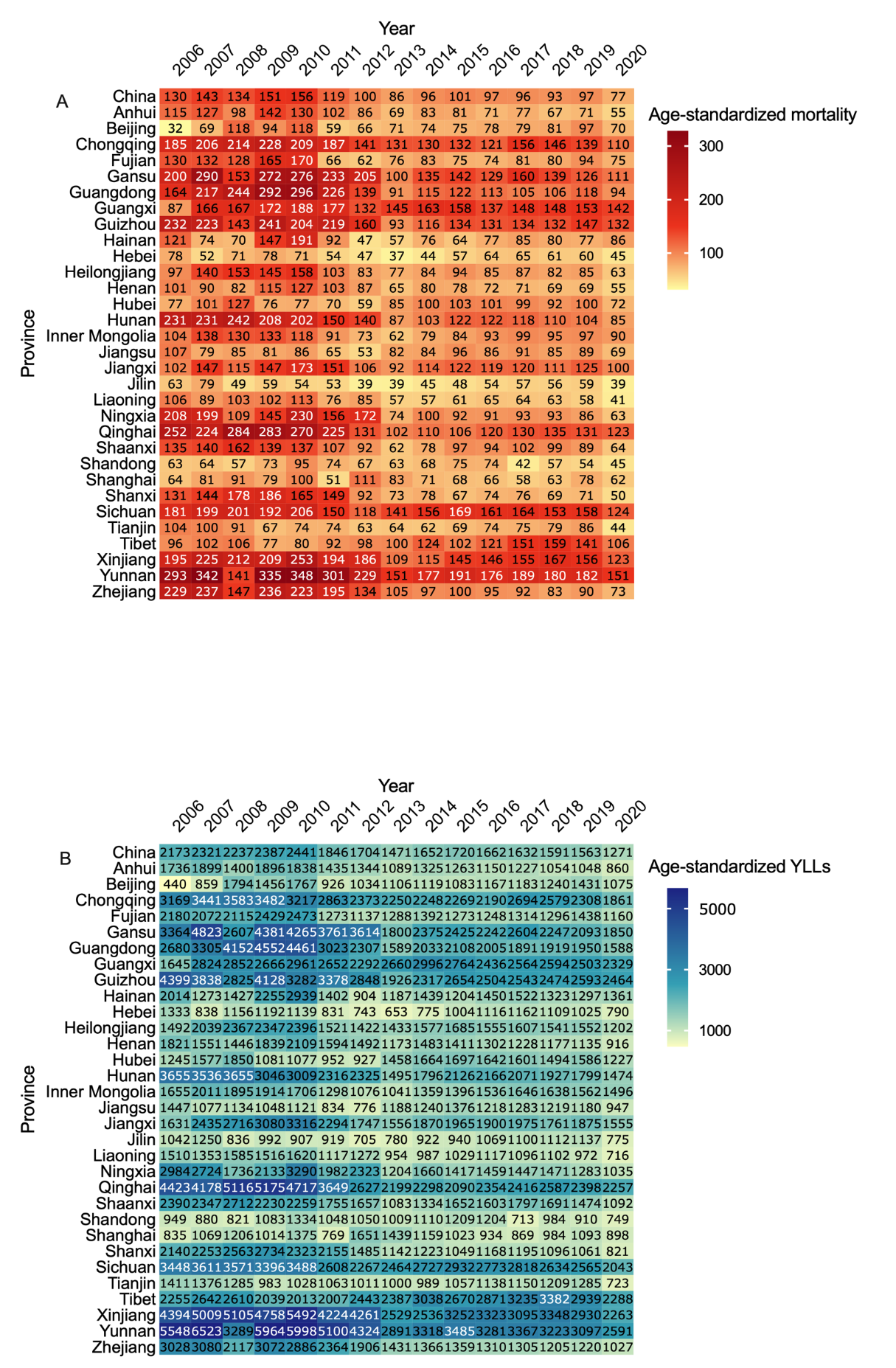
**

**References**

1. Angus DC, Linde-Zwirble WT, Lidicker J, Clermont G, Carcillo J, Pinsky MR. Epidemiology of severe sepsis in the United States: Analysis of incidence, outcome, and associated costs of care. Crit Care Med. 2001;29:1303–10.
2. Weng L, Zeng XY, Yin P, et al. Sepsis-related mortality in China: a descriptive analysis. Intensive Care Med. 2018;44:1071–80.
3. Vos T, Lim SS, Abbafati C, et al. Global burden of 369 diseases and injuries in 204 countries and territories, 1990–2019: a systematic analysis for the Global Burden of Disease Study 2019. Lancet. 2020;396:1204–22.
4. Health Commission of the People’s Republic of China. 2020 Year book of health in the People’s Republic of China. Beijing; 2021.
5. Health Commission of the People’s Republic of China. National Standard Classification and Codes of Diseases. Beijing; 2001.
